# A Large Impact of Obesity on the Disposition of Ivermectin, Moxidectin and Eprinomectin in a Canine Model: Relevance for COVID-19 Patients

**DOI:** 10.3389/fphar.2021.666348

**Published:** 2021-05-20

**Authors:** Alain Bousquet-Mélou, Anne Lespine, Jean-François Sutra, Isabelle Bargues, Pierre-Louis Toutain

**Affiliations:** ^1^ INTHERES, INRAE, ENVT, Université de Toulouse, Toulouse, France; ^2^ The Royal Veterinary College, Hatfield, United Kingdom

**Keywords:** ivermectin, moxidectin, obesity, dosage regimen, canine model, pharmacokinetics, COVID-19

## Abstract

Ivermectin (IVM) and moxidectin (MOX) are used extensively as parasiticides in veterinary medicine. Based on *in vitro* data, IVM has recently been proposed for the prevention and treatment of COVID-19 infection, a condition for which obesity is a major risk factor. In patients, IVM dosage is based on total body weight and there are no recommendations to adjust dosage in obese patients. The objective of this study was to establish, in a canine model, the influence of obesity on the clearance and steady-state volume of distribution of IVM, MOX, and a third analog, eprinomectin (EPR). An experimental model of obesity in dogs was based on a high calorie diet. IVM, MOX, and EPR were administered intravenously, in combination, to a single group of dogs in two circumstances, during a control period and when body weight had been increased by 50%. In obese dogs, clearance, expressed in absolute values (L/day), was not modified for MOX but was reduced for IVM and EPR, compared to the initial control state. However, when scaled by body weight (L/day/kg), plasma clearance was reduced by 55, 42, and 63%, for IVM, MOX and EPR, respectively. In contrast, the steady-state volume of distribution was markedly increased, in absolute values (L), by obesity. For IVM and MOX, this obese dog model suggests that the maintenance doses in the obese subject should be based on lean body weight rather than total weight. On the other hand, the loading dose, when required, should be based on the total body weight of the obese subject.

## Introduction

Ivermectin (IVM) is a broad spectrum macrocyclic anti-parasitic drug, active against internal parasites (nematodes) and ectoparasites (arthropods) (Fox, 2006). It is used in both human and veterinary medicine. It has been recommended for extensive use in humans for prevention of onchocerciasis and to combat river blindness (Cupp et al., 2011). Mass drug administration of IVM is also now proposed as a complementary malaria vector control tool (The Ivermectin Roadmappers, 2020). IVM is used to treat scabies (Elmogy et al., 1999), especially severely crusted scabies lesions in immunocompromized patients or when topical therapy has failed (Fawcett, 2003). The oral dosage of IVM is body-weight-based with a typical recommended anti-parasitic dose of 200 μg/kg (Anonymous, 2020b). This dose rate provides a wide margin of safety (Guzzo et al., 2002). A recent meta-analysis indicated that a dosage of 800 μg/kg was well-tolerated in patients with parasitic infections (Navarro et al., 2020) and more than 2.5 billion doses of IVM have been distributed over the last 30 years (Chaccour et al., 2020).

The avermectins are lipophilic, IVM LogP = 4.4 as also is the structurally related moxidectin (MOX) (LogP = 5.3), with an endectocidal profile similar to that of IVM (Prichard et al., 2012). It has recently been licensed in humans for the treatment of onchocerciasis (Milton et al., 2020), recommended at a single oral dose of 8 mg (Anonymous, 2018). MOX is also a promising drug for treating scabies infection, its long half-life allowing for single-dose treatment, while IVM requires repeated doses (Bernigaud et al., 2016). Obesity is a frequent pathology which can significantly alter the pharmacokinetics of lipophilic drugs (Cheymol, 2000), thus requiring dose adjustments (Knibbe et al., 2015). But so far, no dosage recommendations for IVM and MOX have been proposed in obese patients. This is potentially a major concern, especially for IVM, the dose of which is recommended on the basis of body weight. This lack of data in obese subjects has become notably problematic for a recent, off-label indication for IVM, namely the prevention and treatment of COVID-19 infections.

Recent *in vitro* studies, using kidney-derived cell line Vero-hSLAM cells, demonstrated that IVM has a virucidal action against coronavirus-2 (SARS-CoV-2) (Caly et al., 2020) as well as several other viruses (Heidary and Gharebaghi, 2020). However, chloroquine and hydroxychloroquine, drugs that inhibit the ability of SARS-CoV-2 to infect the kidney-derived cell line Vero, were not efficacious, when using a more relevant test system to assess the entry of SARS-CoV-2 into lung (Hoffmann et al., 2020). MOX has the same antiviral *in vitro* potency as IVM for SARS-CoV-2 (Jan et al., 2021). These data raised the expectation that avermectins, and especially IVM or MOX, might be used in combination with other drugs for the treatment of COVID-19 infections. IVM is currently undergoing assessment in clinical interventional treatment in 45 clinical trials (Anonymous, 2020a) listed in the data base (Clinical trial.gov of the U.S. National Library of Medicine). However, virucidal concentrations *in vitro* (2,000–5,000 nmol/L) were much higher, by several orders of magnitude, than those required for anti-parasitic effects achieved *in vivo*. This led several authors to cast doubt on the potential benefits of systemic IVM administration for prevention or treatment of COVID-19 (Bray et al., 2020; Anonymous, 2021). Alternatively, others have recommended evaluation of high-doses of IVM (Camprubí et al., 2020). Using a modeling approach to describe the time development of viral load in Vero E6 cells, it was shown that IVM (300 and 600 μg/kg q24 h for 3 days) seemed to be at least partially effective on viral load that decreased by 0.3–0.6 log units and exposure by 8.8–22.3%. It was concluded that IVM, 600 μg/kg daily for 3 days (a dosage regimen much higher than the routinely recommended single dose of 200 μg/kg), particularly when given around the time of positivity, may have meaningful impact (Kern et al., 2021). In a non-peer reviewed meta-analyses investigating IVM in randomized clinical trials, it was reported that it was associated with a faster viral clearance than controls, this effect being dose- and treatment duration-dependent (Hill et al., 2021). In the same meta-analysis, also reported was reduced mortality but it was concluded that the optimal dose of IVM is not established. Recently a randomized clinical trial reported that, among adults with mild COVID-19 infection, a 5 days course of ivermectin did not significantly improve the time to resolution of symptoms, compared with placebo, (López-Medina et al., 2021). Consequently, new clinical trials are currently evaluating higher doses, up to 1.2 mg/kg for 5 days. Therefore, it is anticipated that IVM, and also MOX, should be administered using repeated doses significantly higher than those recommended for parasiticidal indications.

Potentially, both IVM and MOX may require contextual adjustments of dose for treatment of COVID-19 infections. Indeed, it is established that obesity is a major risk factor for COVID-19 (Williamson et al., 2020) with higher risks for hospitalization, admission to intensive care units and mortality (Popkin et al., 2020b). Exacerbation of signs and symptoms of COVID-19 results from several mechanisms, including impaired immunity, chronic inflammation and increased proneness to blood clotting (Wadman, 2020b). Another negative effect of obesity is potential disruption of the Blood Brain Barrier (BBB) for which P-glycoprotein (P-gp) is a major efflux transporter (Miller et al., 2008). This has been reported in obese humans and animals fed high fat diets (Rhea et al., 2017). This was not observed in our obese beagle dogs. However, beagle dogs differ substantially from humans, regarding affinity of P-gp for various substrates (Xia et al., 2006). Normally, IVM and MOX have wide safety margins, as they do not penetrate the BBB, due to restriction by the P-glycoprotein (P-gp) efflux transporter (Schinkel et al., 1994; Ménez et al., 2012). However, when the BBB is disrupted, IVM penetration into the brain may be increased, leading to neurotoxicity through drug binding to central GABA-gated receptors (Chandler, 2018; Baudou et al., 2020).

Despite all these reservations and uncertainties on IVM efficacy and the appropriate dosage to provide an antiviral action, if any, IVM is widely used off-label and even approved in certain countries (Vora et al., 2020). It can therefore be anticipated that IVM and MOX, promoted through various media to prevent COVID-19, may be used at unsafe doses, especially in obese patients, in an attempt to achieve *in vivo* the virucidal concentrations obtained *in vitro*.

In this report, the effect of obesity on the disposition of IVM and MOX and additionally on a third avermectin, eprinomectin (EPR) (XLogP3-AA = 3.8) in a canine model of dietary obesity is documented (Rocchini et al., 1987). Whilst EPR is not licensed in humans, it is used extensively in veterinary medicine. It is included in this evaluation, as there is considerable evidence of self-medication and self-dosing with veterinary products in COVID-19 subjects (Momekov and Momekova, 2020). This has led the United States Food and Drug Administration (FDA) to strongly discourage self-medication with avermectins intended for animals (Solomon, 2020). The data used in this report was previously presented as a meeting abstract (Bargues et al., 2009) and as a pharmacy dissertation (Bargues, 2011).

## Materials and Methods

The data generated by Bargues (2011) has been reanalyzed. Individual data (plasma concentrations, body weight, percentage of body fat) are presented in Supplementary Material S1, Supplementary Tables S1–S3). The study was conducted in seven female beagle dogs, aged 2 years and weighing 10.4 ± 0.9 kg at trial commencement. They were housed in pairs in large cages in kennels of the Veterinary School of Toulouse. Each dog received an intravenous bolus of a drug combination, containing 66 μg/kg of each of three drugs, IVM, MOX, and EPR, before (first period, control status) and again after (second period, obese status) 4 months on a high fat diet. Dogs were fed once daily and daily feed consumption recorded. During the control period, dogs were fed a commercial pet chow diet (Croquettes Royal Canin Adulte Medium, Aimargues, France); this provided an energy supply of 3,930 KCal/kg. The food ration (approximately 150 g per dog) was calculated according to the maintenance energy needs with the formula 130 * BW^0.75^ KCal adjusted to maintain a stable weight. For the second period, a dog chow of higher calorific value was provided (Croquettes Eukanuba Puppy Junior Aliment sec, Iams France, Neuilly sur Seine, France) with an energy content of 4,500 KCal/kg. In addition, raw beef fat (8,500 KCal/kg) was given to provide an overall energy feed supply of 6,100 KCal/kg, comprising 60% by the commercial chow and 40% by the beef fat. The objective of doubling the energy content of the ration in the second period was to increase body weight by 40% and to maintain it at this level throughout the second period. The fattening period was of 4 months duration. In both periods, dogs were weighed twice in each week. One adipolysis episode was induced by food restriction at 10 days (D) after administration of the test articles, *i.e.,* between D10 and D15 for the first and the second period and from D26 to D31 only for the second period. For the first 2 days of each of these episodes, dogs were fasted and, for the three subsequent days, they received 50 g (approximately 200 Kcal) of the dog chow used during the control period.

Body Score Condition and body mass indices were measured according to those used to diagnose obesity in dogs in normal veterinary practice (Mawby et al., 2004). The percentage of body fat was evaluated using equations incorporating abdominal circumference and the length of the kneecap-tip of the calcaneus (Bargues, 2011). Body composition was also determined using the deuterium dilution technique for control and obese status. A 99.98% deuterium oxide solution (SigmaR, L’Isle d’Abeau Chesnes, La Verpillière, France) was administered at a dosage of 0.2 g/kg intravenously by catheter in the cephalic vein. Blood samples were obtained from the jugular vein (5 ml into heparinized tube) at times of 15, 30, 60, 90, 120, 150, 180 min post-administration. Plasma was harvested by centrifugation and stored at −80°C prior to analysis. Samples were analyzed for deuterium by mass spectrophotometry at the Aberdeen Center for Energy Regulation and Obesity (Aberdeen) laboratory (Król and Speakman, 1999).

A solution of IVM (Ivomec®, 1% solution for injection for cattle, Merial, France), EPR (Sigma, France), and MOX (Cydectin^®^, solution 1% injection for cattle, Fort Dodge), in a volume of approximately 2 ml, was prepared in an intralipid buffer solution. The buffer solution was prepared from dog serum and a lipid emulsion (intralipid 20%, Fresinius Kabi) 200v/v; this ensured dissolution of the test article *in vivo*.

Administration was via a cephalic vein catheter. The dose rate of each substance was 66 μg/kg. The total dose was 198 μg/kg. The commonly used therapeutic dose of IVM, for treatment of parasitic infections in target species is 200 μg/kg. Blood samples (5 ml) were collected into heparinized tubes by direct puncture from the jugular vein, before administration and at 5, 15, 30 min after administration, then at 1, 2, 4, 8, 12 h and regularly up to 53 days after administration. A further sample, 63 days after administration, was taken in the second period in obese dogs. Samples were centrifuged and plasma frozen at −20°C. The assays of MOX, IVM, EPR and the principal metabolite of IVM, 3-O-demethyl-ivermectin, were conducted using validated HPLC-fluorescence detection methods (Alvinerie et al., 1995; Sutra et al., 1998). The lower limit of quantification for the three analytes was 0.1 ng/L. The coefficients of variations for intra-day precision ranged from 3.0 to 7.8% for MOX and from 0.4 to 9% for IVM and its metabolite. The coefficients of variation for inter-day precision were 5.3% for MOX and 5.7% for IVM and its metabolite.

### Data Analysis

**FIGURE 1:**
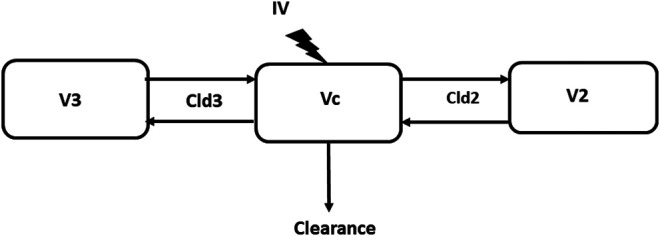
The 3-compartmental model. Vc, V2, and V3 are the volumes of distribution of the central, superficial and deep peripheral compartments, respectively. Cld2 and Cld3 are the distribution clearance for the superficial and deep compartment, respectively.

Pharmacokinetic modeling was carried out using commercially available software (Phoenix NLME version 8.3, Certara, St. Louis, MO, United States). In a first step, each data set for each dog was individually analyzed by non-compartmental analysis (NCA) using the model 200–202, with dose expressed by BW (i.e., 66 μg/kg). In a second step, all pairs of data sets for each test article were analyzed using a Nonlinear Mixed Effects (NLME) approach to generate population pharmacokinetic parameter estimates. For this analysis, the dose was not scaled by BW. Two- and three-compartment models were evaluated to identify the model that best described the data-set. The two models were compared using the likelihood ratio test and the 3-compartment model was selected. Parameterization was in terms of plasma clearance (CL), inter-compartmental clearance(s) (Cld) and volume(s) of distribution (V), with Vc, V2, V3, CL, Cld2, and Cld3 being the primary estimated parameters (Figure 1). The following parameters were computed as secondary parameters, namely the steady-state volume of distribution (Vss) with Vss being the sum of Vc, V2 and V3, the mean residence time (MRT) as the ratio of Vss and clearance and the terminal half-life computed from clearance and volume terms (Dubois et al., 2011).

The between-subject variability (BSV) was modeled using an exponential model, and hence the clearance for the *i*th subject was written as:

$$Cl_{i}=\theta_{median}\times exp\left(\eta_{i}\right)$$ (1)

Where $Cl_{i}$ is the clearance for one of the test article in the *ith* animal$,\theta_{median}$ is the population median clearance (typical value of clearance) and $\eta_{i}$ the deviation (noted ETA) associated with the *ith* animal from the corresponding $\theta_{median}$ population value. Other individual parameters (i.e*.,* Vc, V2, V3 and Cld2, Cld3) were modeled using equations of the same form. The distribution of the ETAs was assumed normal with a mean of 0 and a variance ($\omega_{x}^{2}$). In addition, the individual parameters and consequently their corresponding ETAs can be correlated. All these correlations were estimated and the corresponding covariances were stored in the full variance-covariance omega matrix. The following Eq. 2 was used to convert the variance ($\omega_{clearance}^{2}$) of the log-transformed clearances into a coefficient of variation (CV %) in the original scale:

$$CV_{clearance}\left(\%\right)=100\times \sqrt{exp\left(\omega_{clearance}^{2}\right)-1}$$ (2)

The shrinkage of random effects toward the means was calculated for the ETAs (Savic and Karlsson, 2009) with Eq. 3:

$$shrinkage=1-\frac{SD\left(EBE_{\eta }\right)}{\omega }$$ (3)

Where ω is the estimated variability for the population and SD is the standard deviation of the individual values of the empirical Bayesian estimates (EBE) of η.

The residual model was an additive plus a multiplicative (proportional) model of the form:

$$C\left(t\right)=f\left(\theta ,Time\right)\times \left(1+\varepsilon_{1}\right)+\varepsilon_{2}$$ (4)

With *ε*_1_ and *ε*_2_, the multiplicative and additive error terms having a mean of 0 and a variance noted *σ*_1_ or *σ*_2_, respectively. The additive sigma is reported as its standard deviation with the same units as serum concentration (ng/ml) and the multiplicative sigma as the corresponding coefficient of variation.

Parameter estimation was based on minimizing an objective function value (OFV), using maximum likelihood estimation given for each model. A Laplacian engine was used for analyses approximating the marginal likelihood, while searching for the maximum likelihood. There were no censored data. A bootstrap approach (*n* = 50 samples) was used to estimate typical mean values of parameters and precision of estimates (SE), reported as the corresponding CV%. To evaluate the overall performance of the final model, a Visual Predictive Check was plotted to compare actual observations with simulated replicates from the model (500 replicates per investigated dogs). The 80% prediction intervals (quantiles 10–90%) were constructed and plotted together with the observed data allowing for a visual assessment of the agreement between simulation and observation. Diagnostic plots, the distribution of errors, and the precision of the parameter estimates were used as tools to evaluate the goodness of fit and to compare models.

The pivotal hypothesis of the analysis was that obesity was the covariate able to influence pharmacokinetic parameters and an analysis with the dogs status as covariate (control vs. obese) was carried out to evaluate its significance with (Eq. 5):

$$Param=\theta_{median}\times exp\left(\theta_{1}\times X_{1}\right)$$ (5)

where *Param* is one of the structural parameters of the disposition model (Vc, V2, V3, CL, Cld2, Cld3), *X*_1_ is an indicator variable with a value of 0 for control condition and of 1 for obesity and $\theta_{1}$, the fixed effect of the covariate. For example, for Vc, the model was given either by Eq. 6 for the control condition, or Eq. 7 for the obese condition:

$$Vc=\theta_{Vc_{median}}\times exp\left(\eta Vc\right)$$ (6)

$$Vc=\theta_{Vc_{median}}\times exp\left(\theta_{1}\right)\times exp\left(\eta Vc\right)$$ (7)

where $\theta_{VC_{median}}$ is the typical value of Vc in the control condition, ηVc is the ETAs associated with Vc and $\theta_{1},$the fixed effect of the covariate for the obesity condition. If $\theta_{1}$ is significantly different from zero, it provides evidence that a difference exists between the control and obese condition for Vc. No attempt was made to explore other covariates.

As there was a single covariate, the Phoenix Shotgun approach was used to evaluate all 64 possible scenarios (combination of parameters influenced or not by the covariate) to rank them using the Bayesian information criterion (BIC). A step-wize covariate search mode was also used to define the statistical significance of the covariate for each of the structural parameters of the model. This run mode performs a step-wize forward or backward addition or deletion of covariate effects (by adding/deleting one at a time) to determine the improvement of the final model based on the BIC. For the present analysis, we selected a BIC value of 6.635 for adding a covariate and a value of 10.823 for deleting a covariate, as these values are equivalent to *p* < 0.01 and *p* < 0.001 for minus twice the log-likelihood (2-LL) criterion when using the LRT test (Hutmacher and Kowalski, 2015).

## Results

**FIGURE 2:**
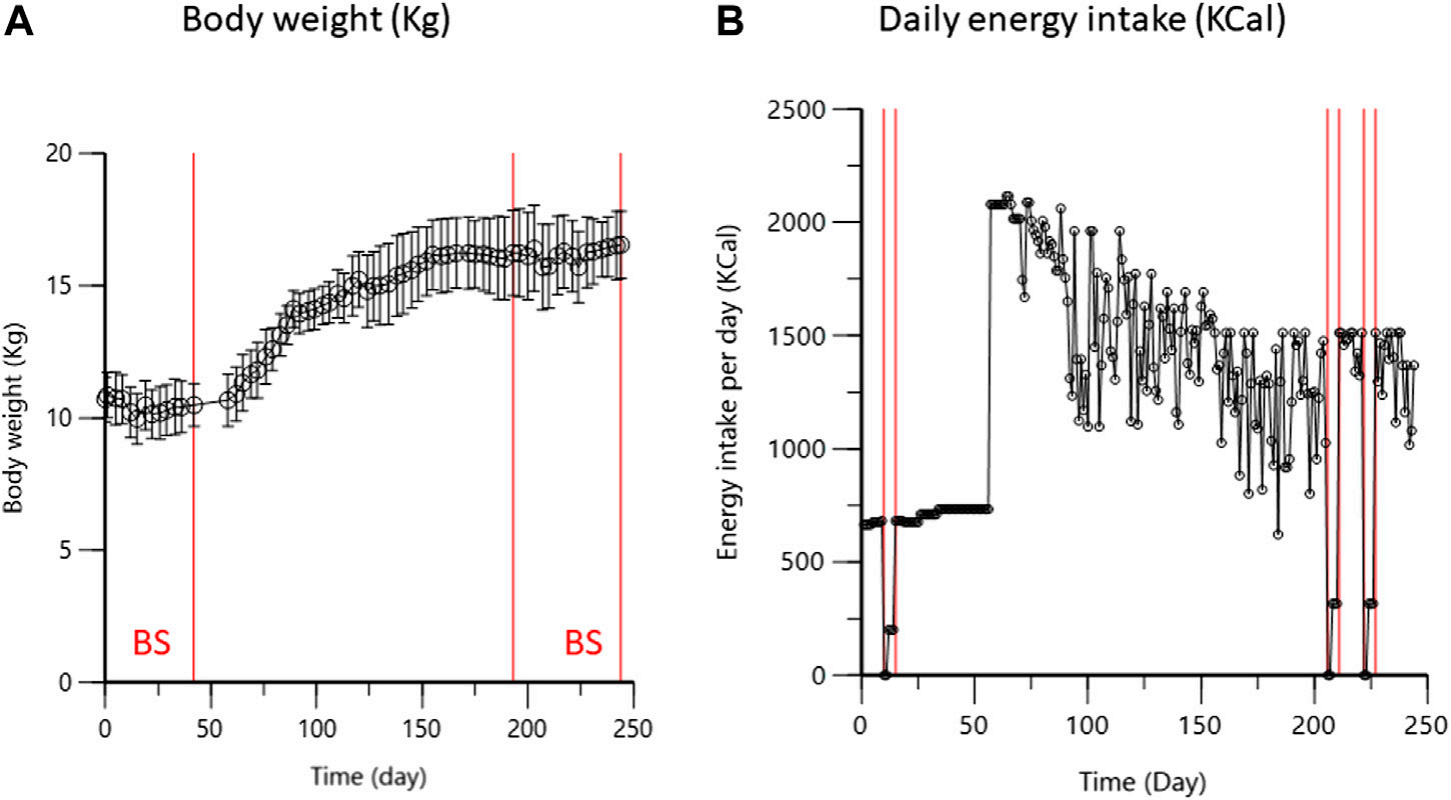
**(A)**: Time development (days) of body weight (kg) (mean and SD) for the seven dogs. Red vertical lines indicate time of blood sampling (BS) during the first (lean) and second period (obese); **(B)**: average daily caloric intake (Kcal) for the seven dogs during the study. Red vertical lines indicate episode of energy intake restriction (10–15, 206–211, and 222–227 days).

Figure 2 depicts the time development of the average BW (kg) and caloric intake for the seven dogs. During the first period, the average BW was 10.4 ± 0.9 kg (min-max: 8.1–12.1 kg) and the energy requirements, maintaining this stable control BW, amounted to approximately 750 Kcal/day. The fattening ration provided excess caloric intake throughout the duration of the high fat diet. When the weight stabilization phase was reached (approximately 100 days after the start of fattening, i.e., on D150), the percentage weight gain was 57 ± 25% (*p* < 0.01). The obesity status, defined as 20% weight gain over normal weight, was largely achieved. As during the first blood sampling period, BW of the dogs was stable during the second sampling period, ranging from to 15.2 ± 1.7 kg (min-max: 13.6–18.9 kg).

The percentages of body fat (mean and SD) calculated from the body mass index, during the first and second periods, were 24.6 ± 4.6 and 38.2 ± 2.6%, respectively (*p* < 0.01). Using the deuterium oxide dilution technique, the average body fat percentage was 21.9 ± 3.3% (range 15.9–23.8%) in the first period and 43.7 ± 2.3% (range 39.9–46.1%) in the second (*p* < 0.01). The high fat diet produced an increase in body fat percentage of 104 ± 41%.

**FIGURE 3:**
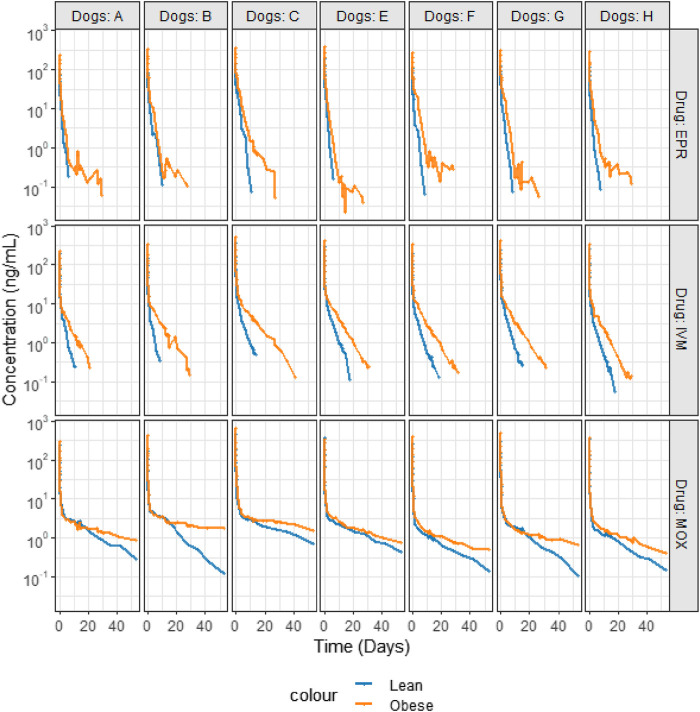
Semi-logarithmic plots of the disposition curves of IVM, MOX, and EPR after a single administration of each drug as a cocktail at the dose rate of 66 μg/kg by IV injection in seven dogs in control (blue curves) and obesity (orange curves) conditions.

Individual plots for each test article and each dog, before and after, fattening are depicted in Figure 3. Visual inspection indicates that obesity exerted a large effect on the disposition of IVM, MOX, and EPR, with much slower elimination for each test article during the period of obesity.

### Non-Compartmental Analysis

Results of the NCA are presented in Table 1.

**TABLE 1:** Results of the NCA analysis (Model 200–202, Log-linear trapezoidal rule) for the three drugs and seven dogs.

Parameters (units)	Substance	Status	Mean	SD	Variation (%)	*p* value
Clearance (ml/kg/day)	IVM	Lean	1,290	393		
	IVM	Obese	583	178	−55%	0.001
	MOX	Lean	748	249		
	MOX	Obese	431	174	−42%	0.001
	EPR	Lean	1,503	387		
	EPR	Obese	552	159	−63%	0.0001
Vss (ml/kg)	IVM	Lean	2,951	583		
	IVM	Obese	3,124	546	+6%	NS
	MOX	Lean	10,917	2,705		
	MOX	Obese	15,079	2,772	+38%	0.0171
	EPR	Lean	1,751	388		
	EPR	Obese	1,246	341	−29%	0.0190
MRT (day)	IVM	Lean	2.38	0.51		
	IVM	Obese	5.57	0.95	+134%	0.001
	MOX	Lean	15.40	4.23		
	MOX	Obese	40.62	20.56	+164%	0.027
	EPR	Lean	1.21	0.30		
	EPR	Obese	2.31	0.46	+91%	0.001
Half-life (day)	IVM	Lean	2.47	0.75		
	IVM	Obese	4.36	0.37	+76%	0.032
	MOX	Lean	13.68	3.45		
	MOX	Obese	35.63	13.71	+161%	0.013
	EPR	Lean	0.99	0.16		
	EPR	Obese	3.03	1.05	+206%	0.0029
Vz (ml/kg)	IVM	Lean	4,508	1,525		
	IVM	Obese	3,637	1,044	−19%	NS
	MOX	Lean	14,471	5,357		
	MOX	Obese	19,924	4,494	+38%	0.015
	EPR	Lean	2,140	662		
	EPR	Obese	2,426	1,084	+13%	NS

Clearance, plasma clearance; Vss, steady-state volume of distribution; MRT, mean residence time computed with extrapolation to infinity. Half-life, terminal half-life; Vz, Volume of distribution associated with the terminal phase. *p* values obtained with a paired *t* test.

For the three test articles, plasma clearance, expressed per kg BW, was significantly decreased (by 55, 42, and 63% for IVM, MOX, and EPR, respectively) during the obesity period. This was associated with large increases in MRT (134, 164, and 91% for IVM, MOX, and EPR, respectively) and terminal half-life (76, 161, and 206% for IVM, MOX, and EPR, respectively) For volume of distribution, there was no significant difference for IVM, an increase for MOX (38%) and a decrease for EPR (29%). Similarly, for Vz (i.e., Varea) a parameter associated with the terminal phase, there were no differences for IVM and EPR, while it was significantly increased by MOX (38%) *p* = 0.015.

**FIGURE 4:**
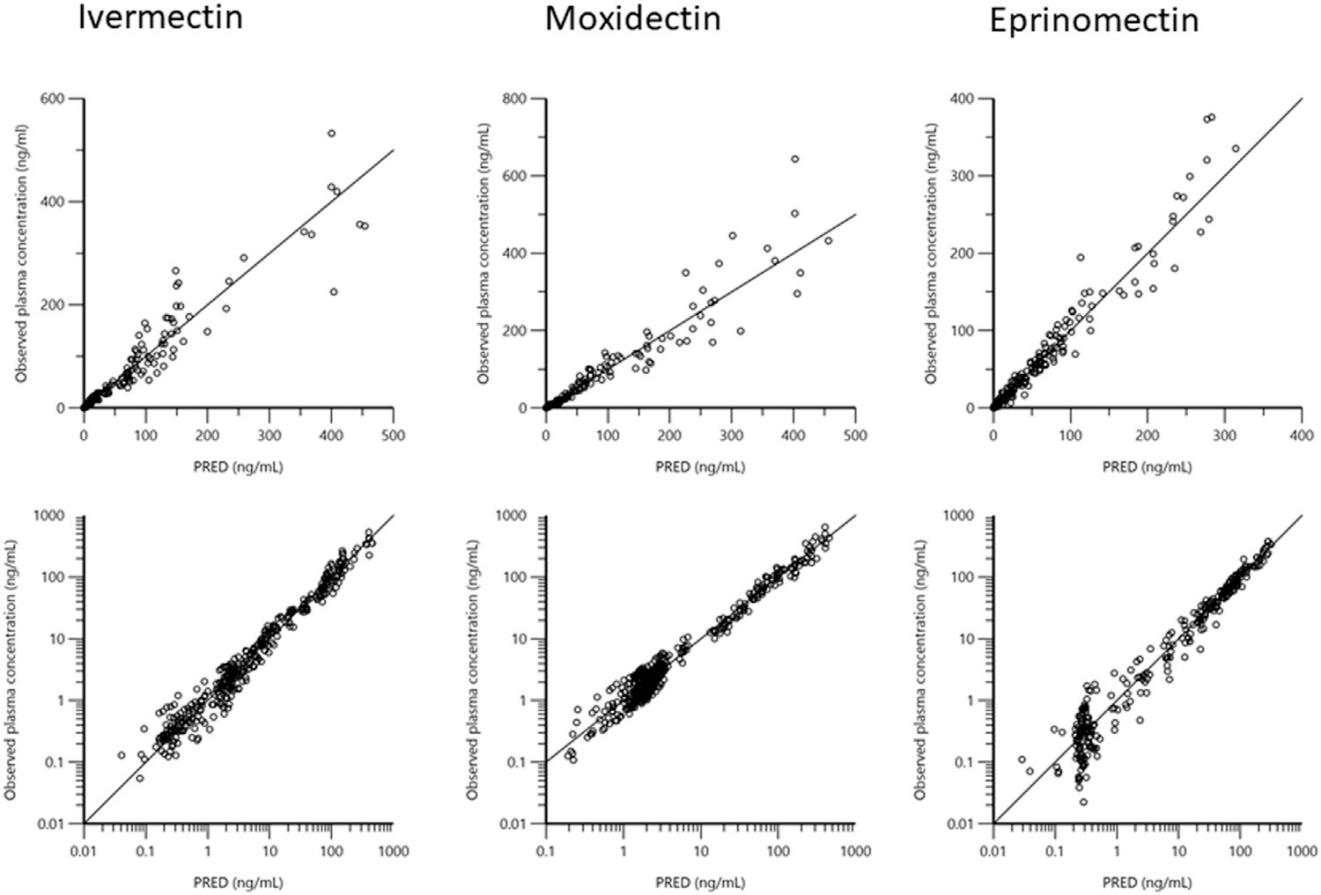
Plots of the dependent variable i.e. of observed plasma concentrations (ng/mL) vs. population predicted plasma concentrations (PRED) (no random component) for the three drugs. The plots illustrate observed vs. fitted values of the model function. Ideally they should fall close to the line of unity y = *x*. Arithmetic scale **(upper)** and logarithmic scale **(lower)**. For both arithmetic and logarithmic scales, data are evenly distributed about the line of identity, indicating no major bias in the population component of the model.

**FIGURE 5:**
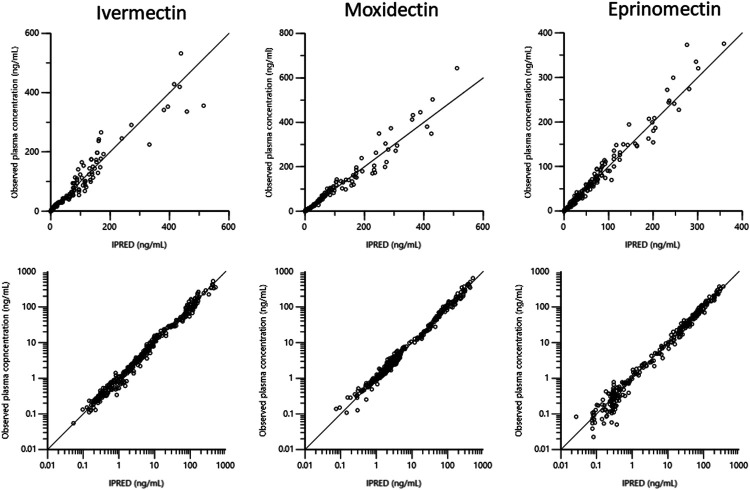
Plots of the dependent variable, observed plasma concentrations (ng/mL), vs. individual predicted plasma concentrations (IPRED) for the three drugs. Individual predictions were obtained by setting random effects to the “*post hoc*” or Empirical Bayesian Estimate of the random effects for the individual dog, from which the plasma concentration observation was made. Thus, the plot illustrates observed vs. fitted values of the model function. Ideally, they should fall close to the line of unity y = *x*. Arithmetic scale **(upper)** and logarithmic scale **(lower)**.

**FIGURE 6:**
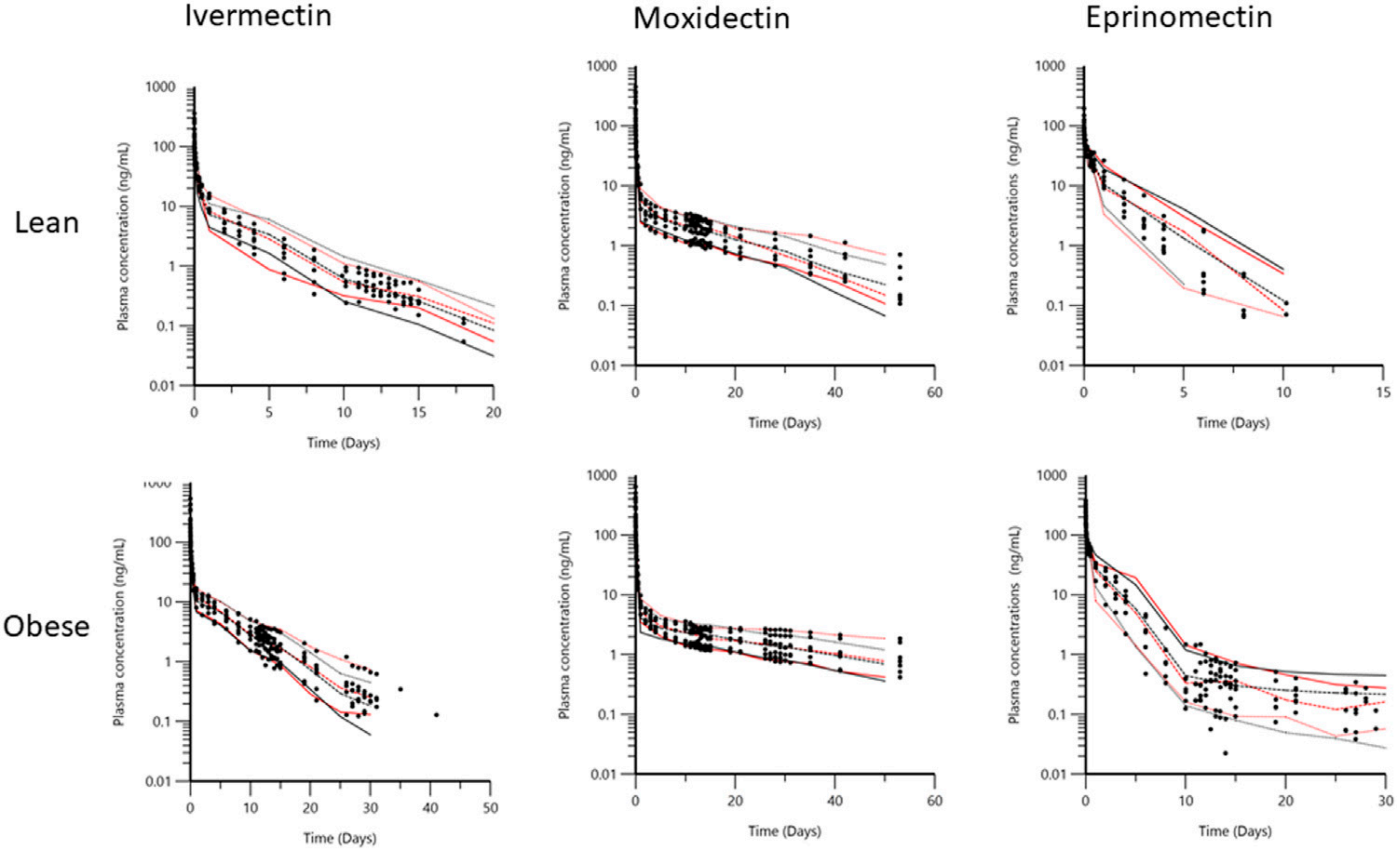
Visual Predictive Check (VPC) obtained with 500 replicates of each dog and each status (lean, obese). For each stratification, the observed quantiles (10, 50, and 90%) were well super-imposed with the corresponding predictive check quantiles over the observed data. Red lines: observed quantiles; Black lines: predicted quantiles; Black symbols: observed data.

In a second step, a compartmental analysis, using a 3-compartmental approach, was used. Figures 4–6 are Goodness-of-fit (GOF) (Observed data vs. population predictions and observed data vs. individual predictions plots) supporting the 3-compartmental structural model, the exponential model for the random component and the additive plus multiplicative model for the error sub-model used to analyze the data.

The adequacy of the 3-compartmental was checked by plotting the Visual Predictive Check (VPC). The 10th, 50th, and 90th percentiles of the simulated distribution were compared to the observations. A binning option (explicit center) was used. VPC from 0 to 2 days is presented in Supplementary Material S3, Supplementary Figures S1–S3. Individual fittings are given in Supplementary Material S3, Supplementary Figures S4–S6. Conditional Weighted Residual values (CWRES) vs. time (Days) are given in Supplementary Material S3, Supplementary Figure S7.

Typical values of the primary structural parameters of the model (thetas), the secondary parameters (MRT, Vss, half-life….), their associated CV% and the SD of the residuals are presented in Tables 2, 3.

**TABLE 2:** Population primary parameters as obtained with a 3-compartment model with covariate (COV) (lean vs. obese); estimates bootstrap (mean and CV%).

Parameters	Units	IVM		MOX		EPR	
		Mean	CV%	Mean	CV%	Mean	CV%
tvVc (lean)	L	0.170	23.55	2.237	7.54	6.546	6.03
tvV2 (lean)	L	4.59	5.42	6.42	4.85	10.40	2.87
tvV3 (lean)	L	26.35	5.88	104.63	11.72	1.36	27.55
tvCl (lean)	L/Day	12.10	7.72	7.84	12.97	14.90	7.44
tvCld2 (lean)	L/Day	125.2	13.30	116.0	7.79	89.9	7.35
tvCld3 (lean)	L/Day	21.31	2.93	22.72	7.30	0.79	22.32
COV Cl	Scalar	−0.266	18.78	0	NC	−0.791	15.43
COV Cld3	Scalar	0	NC	0.161	27.58	0.943	1.54
COV Vc	Scalar	0	NC	0	NC	−0.572	11.07
COV V3	Scalar	0.502	16.49	0.683	22.65	5.026	10.37
tvV3 (obese)	L	43.55	3.87	207.2	6.91	206.8	0.30
tvCl (obese)	L/Day	9.27	10.60	7.84	12.97	6.75	18.54
Error multiplicative	CV%	20.77	8.70	16.51	3.72	18.54	9.39
Error additive (stdev0)	ng/mL	0.001	NC	0.065	55.51	0.108	19.27

Vc, volume of the central compartment; V2, volume of the shallow peripheral compartment; V3, volume of the deep peripheral compartment, Cl, plasma clearance; Cld2 and Cld3, distribution clearance for the shallow and deep compartment; multiplicative component of the error model is expressed as CV% and the additive component of the residual error model by its standard deviation. tv lean, typical values for the control status (lean); COV are the estimate of the fixed effect for covariates (exponential model). tv obese are typical value for the obese status; it is obtained by the product of the tv lean by the exponential of the corresponding scalar (e.g. the tv of clearance for IVM for obese condition is 12.10 L/day fold exp (−0.266) equal to 9.27 L/day. For EPR, lower and upper bounds were used for the bootstrap estimation to prevent spurious estimates from some bootstrap samples, and results (especially precision of estimates) should be considered with caution The average BW was 10.4 ± 0.9 kg (min-max: 8.1–12.1 kg) during the lean period vs. 15.2 ± 1.7 kg (min-max: 13.6–18.9 kg) during the obesity period.

**TABLE 3:** Population secondary parameters obtained with a 3-compartments model with covariate (COV) (lean vs. obese); estimates were obtained from typical values of primary parameters of Table 2.

Parameters	Units	IVM	MOX	EPR
Vss (lean)	L	31.11	113.28	18.30
Vss (obese)	L	48.31	215.89	220.87
MRT (lean)	Day	2.57	14.45	1.23
MRT (obese)	Day	5.21	27.54	32.71
HL (lean)	Day	2.55	12.98	1.35
HL (obese)	Day	4.93	25.22	202.35

Vss, steady-state volume of distribution; MRT, Mean Residence Time (MRT); HL, terminal Half-life. For HL, the calculated parameters for obese status were poorly estimated in terms of precision and the figures for this status should be viewed with caution. For EPR, results should be considered with caution (see comment in Table 2). The average BW was 10.4 ± 0.9* *kg (min-max: 8.1–12.1* *kg) during the lean period vs. 15.2 ± 1.7* *kg (min-max: 13.6–18.9* *kg) during the obesity period.

Data in Table 2 indicate the bootstrap estimates of the parameters (see Supplementary Material S2). Supplementary Table S4 presents all bootstrap results and details how parameters were estimated using either bootstrap or a single run with the seven dogs and corresponding Supplementary Table S5 presents the full omega matrix and shrinkage). Table 2 indicates that clearances, expressed in absolute values, were either not significantly modified (MOX) or even reduced in obese dogs (IVM and EPR). The volume of the deep compartment (V3) was increased for the three drugs. For EPR, lower and upper bounds were used for the bootstrap estimation to prevent spurious estimates from some bootstrap samples and results (especially precision of estimates) should be interpreted with caution.

Inspection of Table 3 shows that Vss was significantly increased in the obesity condition for the three drugs, accounting for the corresponding increase in MRT.

The between-subject variability (BSV) for clearance was 18.11, 28.15, and 21.08%, respectively, for IVM, MOX and EPR. For V3, the deep compartment, BSV was relatively small for IVM and MOX (8.03 and 8.31%) but very high for EPR (149.5%). This was due to the fact that the third phase was not clearly identified in all dogs (see Figure 2). The full OMEGA matrix, the BSV for all parameters and shrinkage are given in Supplementary Material S2, Supplementary Table S5.

**FIGURE 7:**
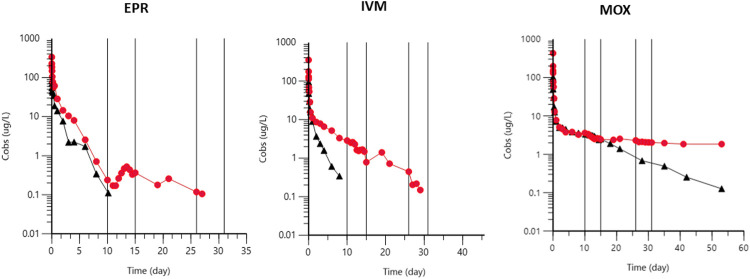
Effect of a 2-day fasting episode followed by a 3-days caloric restriction triggered 10 days (lean dog, black dots) or 10 and 26 days (obese dog, red dots) after administration of EPR, IVM, and MOX on plasma concentrations of each drug in a representative dog (dog B). Vertical lines indicate episodes of fasting (2 days) followed by caloric restriction (3 days).

In the present experiment, we induced in dogs a first episode of fasting (2 days) followed by 3 days of restriction of energy intake 10 days after drug administration and, only during the obesity status, a second fasting episode 26 days after drug administration. This protocol was designed to investigate the effects of lipomobilization on plasma concentrations of the three drugs studied. A clear rebound was obtained only for EPR during the first episode of fasting and only in obese dogs. No such rebound occurred with IVM and MOX (Figure 7).

## Discussion

Conditions of overweight/obesity in humans occur with a prevalence greater than 20% in almost all countries (Popkin et al., 2020b; Popkin et al., 2020a). Currently, 32% of people in the United States are overweight (Wadman, 2020a). Obesity is a classical co-morbid factor for several diseases, including hypertension, cardiovascular disease, dyslipidemia, type-2 diabetes (Khaodhiar et al., 1999) and it was also recently reported for COVID-19. Of almost 17,000 patients hospitalized in United States with COVID-19, were either overweight (29%) or obese (48%) (Chawla et al., 2020). IVM is widely used worldwide and the administrated dose is usually based on patient body weight. The lack of specific dosing guidelines for this drug in obese subjects is partly attributable to the *a priori* exclusion of obese subjects from clinical trials (Han et al., 2007). Given the attention paid recently to IVM in the prevention and treatment of COVID-19 and in view of its lipophilic nature, the present study provides some preliminary data on which to base possible adaptation of dosage in obese patients in general, and in particular those affected with COVID-19. The most appropriate way to address this question would be to conduct population pharmacokinetic studies in the target patients receiving IVM or MOX. However, the results of such studies are not currently available, yet there is current urgency deriving from the COVID-19 situation worldwide. Moreover, there are no universal guidelines for adjusting dosages in cases of obesity (Green and Duffull, 2004). Data from the model used in this study of obesity in dogs provide initial first steps toward more definitive answers.

The dog as a species provides a good comparative model for human obesity, since clinical signs are similar in the two species (Osto and Lutz, 2015). The obesity model used in this study was initially developed to study hypertension (Rocchini et al., 1987; Verwaerde et al., 1999) and it has been used also in pharmacokinetic investigations, because of its ability to rapidly achieve relatively severe obesity and its reversibility. The model has several similarities with human obesity as occurring in hyperinsulinemia and insulin resistance (Rocchini et al., 1987).

The experimental design has enabled use of the same dogs to study the two conditions, control and obese, and the combination/simultaneous drug dosing schedule ensured good discriminating power. The design also allowed comparison of both the influence of obesity on the disposition of the three investigated drugs and generated data indicating differences between them, each drug having its own unique physico-chemical properties. The study also minimized the numbers of animals used experimentally. For IVM and MOX, the data generated for control dogs was in agreement with previously reported findings for IVM (Lo et al., 1985) and MOX (Lallemand et al., 2007). In the latter studies, each drug was administered alone, and this validates drug combination dosing, as indeed it has also been validated for many other compounds (He et al., 2008).

The principal finding from this study is that, in obese dogs, the clearance of the three investigated drugs, expressed in absolute values (L/day), was either not modified (MOX) or reduced (IVM and EPR). The consequence was a significant decrease in clearance when scaled by actual body weight (-55, -42, and -63% for IVM, MOX, and EPR, respectively). This is in line, at least for MOX, with previous reports which demonstrated that the clearance (expressed in absolute value) of several drugs, including phenazone, carbamazepine, lithium, remifentanil, cefazolin and theophylline, was not influenced by obesity (Mahmood, 2012).

In human medicine, and according to WHO recommendations (Anonymous, 2021), ivermectin, for the treatment of onchocerciasis, is typically administered annually as a single dose adjusted for body weight (150–200 μg/kg). On the other hand, for the treatment of COVID-19, repeated doses have also been used. For example, in a controlled clinical trial patients were randomized to receive either ivermectin, 300 μg/kg of body weight per day for 5 days, or placebo (López-Medina et al., 2021). Recommendation of a weight-based oral dosage of IVM assumes that plasma clearance is directly proportional to Total Body Weight (TBW), regardless of body composition. This is supported by several population pharmacokinetic investigations, which have shown that body weight is the relevant covariate (Schulz et al., 2019; Gwee et al., 2020). In a small, homogeneous trial, which enrolled 12 healthy volunteers, only BW was a significant covariate for plasma clearance and volume of distribution, while the Body Mass Index (from 18.1 to 26.4 kg/square meter) was not significant (Duthaler et al., 2019). This is apparently not in line with the present results but, as pointed by others, BMI is a poor predictor of percentage of body fat, especially when the BMI is lower than 27 kg/square meter (Meeuwsen et al., 2010). In practice, this means that the same dose rate will be administered to all patients having the same TBW, whatever their percentage of body fat. More relevant and fully supporting results of the present trial, in a 3-period clinical trial, with 54 healthy adult volunteers compared sequentially, a fixed-dose strategy of 18 and 36 mg single dose regimens was used, based on weight 150-200 μg/kg (Muñoz et al., 2018). It was shown that individuals with high BMI and BW presented higher V/F and terminal half-life. In contrast, no significant association was found between BW and BMI for Cmax and AUC leading the authors to propose the use of fixed dosage regimens rather than the current weight based strategy (Muñoz et al., 2018) Assuming that obesity does not alter the oral bioavailability (Hanley et al., 2010; Knibbe et al., 2015), the present trial also suggests that, in obese subjects, the actual BW should not be considered in computing a maintenance dosage for IVM or MOX. Indeed, clearance and bioavailability are the only pharmacokinetic parameters controlling internal exposure, and the total clearance of the three drugs reported in this study was unchanged or even decreased in obesity, compared to clearance in lean animals. It is concluded that the same total dose should be considered to lean and obese subjects, regardless of their actual BW and dose should be computed on a Lean Body Weight (LBW) basis, not a TBW. We recently reported the case of a patient treated for scabies having a BMI of 53.3 kg/m^2^ and a BW of 158 kg, for which a IVM dose of 114 μg/kg (half the recommended dose) achieved IVM plasma concentrations similar to those reported in normal patients with a dose of 200 μg/kg (Mellon et al., 2019). This is also supported by conclusions reached by others, namely that LBW suffices to explain the influence of body composition on clearance and can therefore adequately predict drug exposure in the obese subjects (Han et al., 2007). The underlying rationale is that 99% of the body’s metabolic processes (including clearance) takes place in lean tissues (Han et al., 2007).

An additional finding of clinical significance is the large increase in the absolute value of volume of distribution (L) in obesity especially that of the deep compartment (V3), as evidenced by compartmental analysis. This supports the hypothesis that V3 represents the adipose tissue, for which IVM, MOX, and EPR display a large affinity. This results in increased MRT and terminal half-life, because these two time parameters are hybrids; they depend on both clearance and volume of distribution (Vss for MRT, Varea or Vz for half-life) (Toutain and Bousquet-Melou, 2004). The practical consequence is a possible greater accumulation of the drugs, with repeated administrations and a longer lag-time to reach a state of equilibrium ensuring the same internal exposure as for the lean counterpart. The delay is approximately 3-fold the terminal half-life (and MRT) and it is increased 2-fold in obesity for IVM and MOX. This leads to long delays from some 10 to 20 days for IVM and from 2 to 4 weeks for MOX in lean vs. obese subjects, respectively.

Given the length of these delays, and if rapid attainment of maximal effect is required, a loading dose could be considered and, for this, the relevant pharmacokinetic parameter is Vss. The absolute value of the latter is doubled in obese subjects for both IVM and MOX. Therefore, the loading dose, for the same plasma concentrations at steady state, must be 2-fold greater in obese than in lean subjects, while the maintenance dose should be unchanged. Comparison of the weight-normalized circumstance, between obese and non-obese individuals, provides insights into how a drug distributes into excess weight (Hanley et al., 2010). When volume of distribution normalized by TBW is similar in obese and non-obese subjects, as in this study, it can be concluded that the drugs exhibit marked sequestration in adipose tissue. Hence, a weight-based loading dose for such a drug is appropriate (Hanley et al., 2010). The present data are consistent with the opinion of Green and Duffull that, according to most published studies, TBW is the best descriptor of volume of distribution in obese subjects (Green and Duffull, 2004). Considering the numerical value of plasma clearance and Vss, it seems that, for a given therapeutic objective, the loading dose for MOX should be much higher than the maintenance dose. This is less the case for IVM. Therefore, it is likely that, if repeated doses are required, and all things being equal in terms of therapeutic objective, dose and dosing interval, IVM is a more convenient therapeutic choice than MOX.

COVID-19 is associated with clinically significant weight loss (Di Filippo et al., 2020) and, in the present experiment, a period of fasting (2 days) was followed by 3 days of restriction of energy intake to ascertain the effects of lipomobilization on plasma concentrations of the drugs studied. A rebound phenomenon occurred for EPR in the obesity condition. On the other hand, this was less marked for IVM and absent for MOX.

With only seven healthy dogs studied intravenously and an experimental model of obesity, we do not claim to have reproduced fully the complexity of the COVID-19 condition. Nevertheless, both the homogeneity and magnitude of the altered disposition obtained in this study, for the three investigated macrocyclic lactones, provide a strong signal to be taken into account in the clinical setting of COVID-19, and beyond that for all those conditions justifying the administration of IVM or MOX in obese subjects.

In conclusion, the present analysis suggests that, when daily dosing is required, the maintenance doses of IVM and MOX should not be adjusted for body weight in obese subjects; dosage should be based on LBW. On the other hand, determining a loading dose must take into account the actual BW and this loading dose will be significantly higher than the maintenance daily dose, especially for MOX, which makes MOX less attractive than IVM in case of repeated dosing. EPR, an avermectin not licensed for use in human medicine, behaves like IVM and offers no specific advantage over IVM and its off-label use in human medicine should be discouraged.

## Data Availability

The original contributions presented in the study are included in the article/Supplementary Material, further inquiries can be directed to the corresponding author.
