# Supplementary material for: A Large Impact of Obesity on the Disposition of Ivermectin, Moxidectin and Eprinomectin in a Canine Model: Relevance for COVID-19 Patients
Source: Front Pharmacol. 2021 May 20;12:666348. doi: 10.3389/fphar.2021.666348 (PMC8173197; doi:10.3389/fphar.2021.666348)
Supplement: Supplementary file 1 [file datasheet3.docx]

**Supplementary material**

**Supplementary file 4**

# **A** l**arge impact of obesity on the disposition of ivermectin, moxidectin and eprinomectin in a canine model: relevance for COVID-19 patients**

**Running title:** obesity and pharmacokinetics of ivermectin, moxidectin and eprinomectin

**Authors:**

A. Bousquet-Mélou^1^, A Lespine^1^, J-F Sutra^1^, I Bargues^1^, P-L. Toutain^1,3^

^1^ *INTHERES, Université de Toulouse, INRAE, ENVT, Toulouse, France.*

^3^ *The Royal Veterinary College, Hawkshead Campus, Hatfield, Herts., AL9 7TA, United Kingdom*

**Orcid numbers**

Bousquet-Melou***:*** https://orcid.org/0000-0002-7661-4311

P-L Toutain: http://orcid.org/0000-0002-8846-8892

***Corresponding author*: Pierre-Louis Toutain**

**Supplementary figures S1, S2 and S3**

Visual Predictive Check (VPC) was obtained with 500 replicates for each dog. For each stratification (lean vs. obese) the observed quantiles (10, 50 and 90%) should be well super-imposed with the corresponding predictive check quantiles over the observed data. Red lines: observed quantiles; Black lines: predicted quantiles; Black symbols: observed data.

**Supplementary Figure S1: Visual Predictive Check (VPC) for the first two days for Ivermectin**

**A: Lean dogs**

**
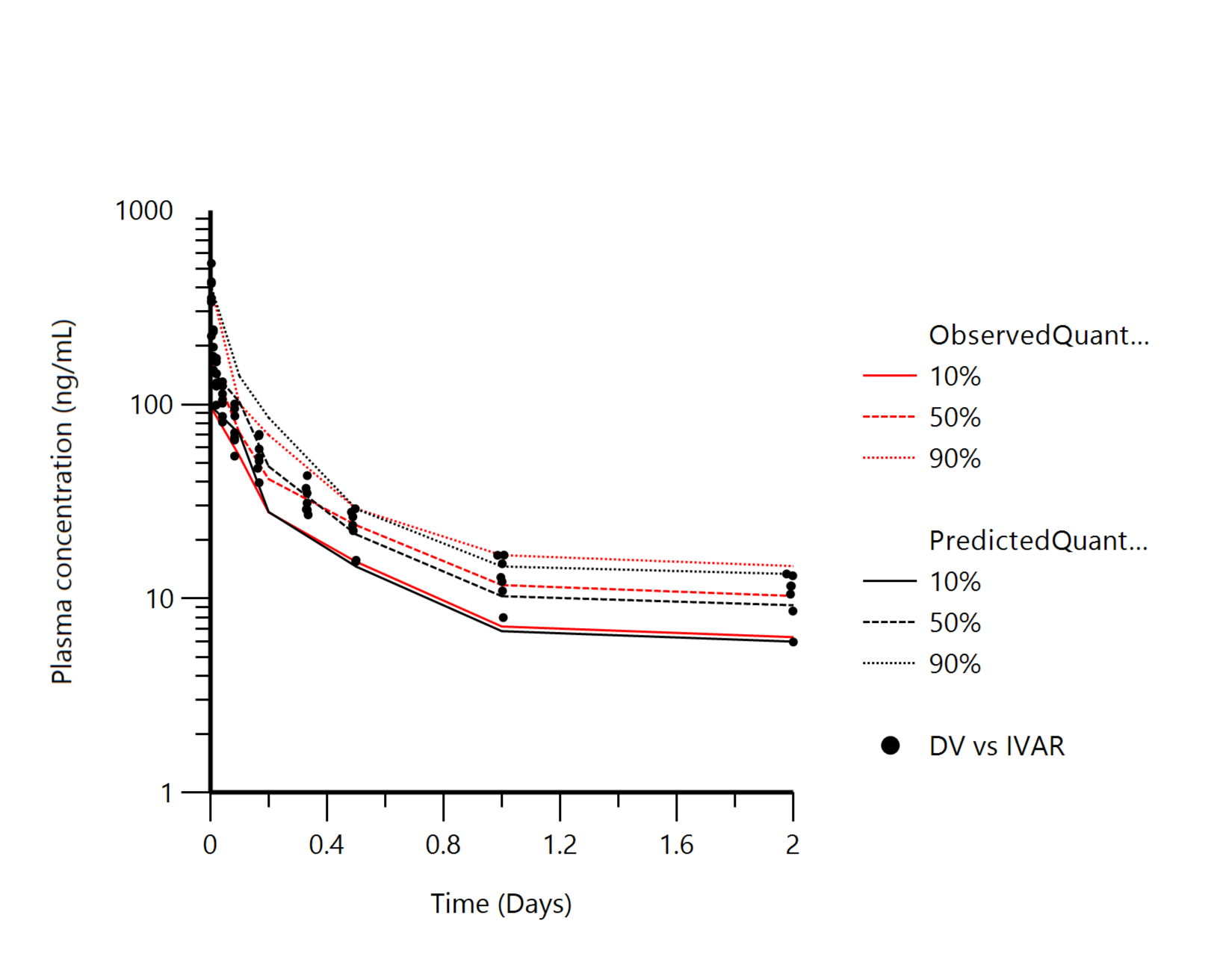
**

**B: obese dogs
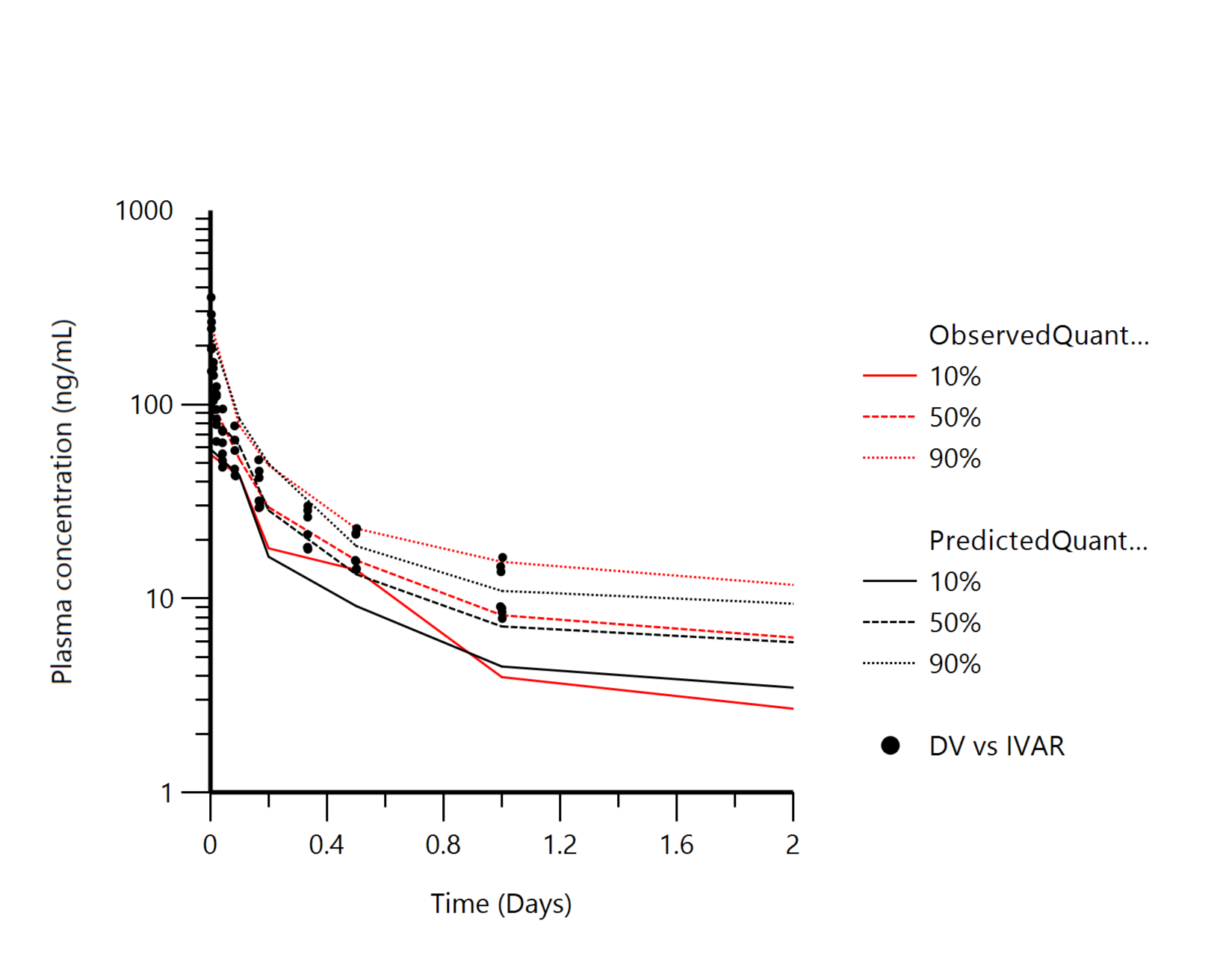
**

**Supplementary Figure S2: Visual Predictive Check for the first two days for Moxidectin**

**A: lean dogs**

**
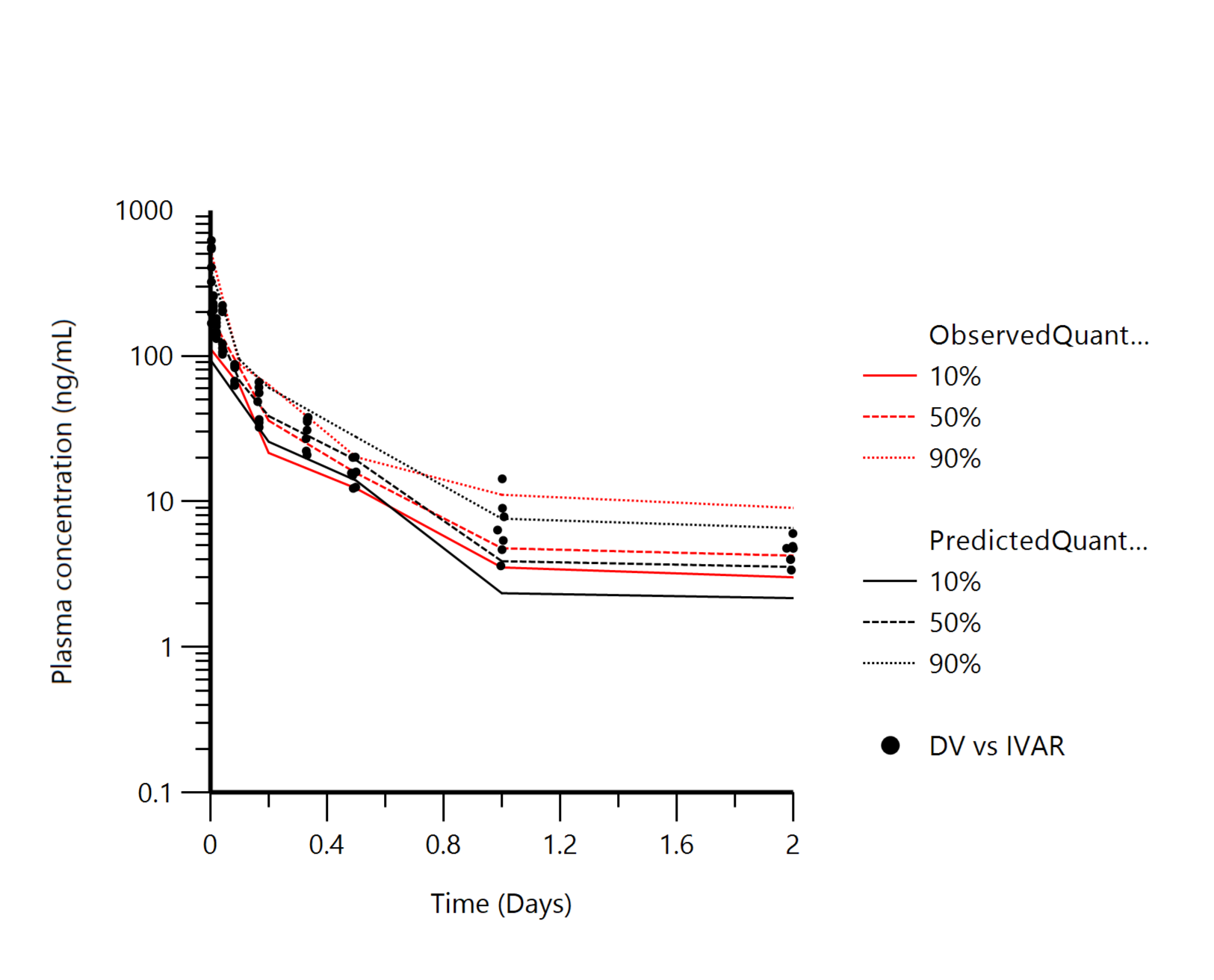
**

**B: Obese dogs
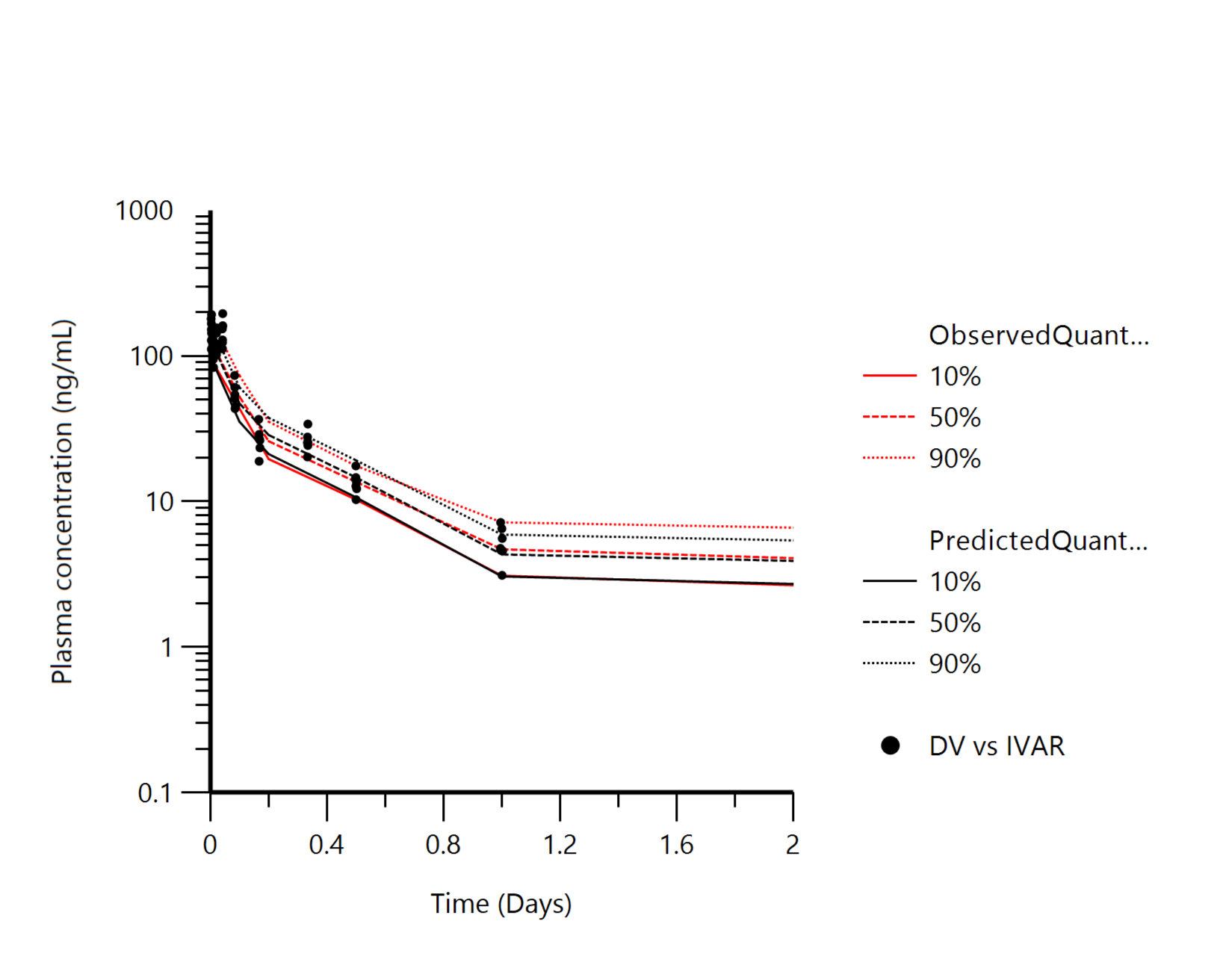
**

**Supplementary Figure S3: Visual Predictive Check for the first two days for Eprinomectin**

**A: lean dogs**

**
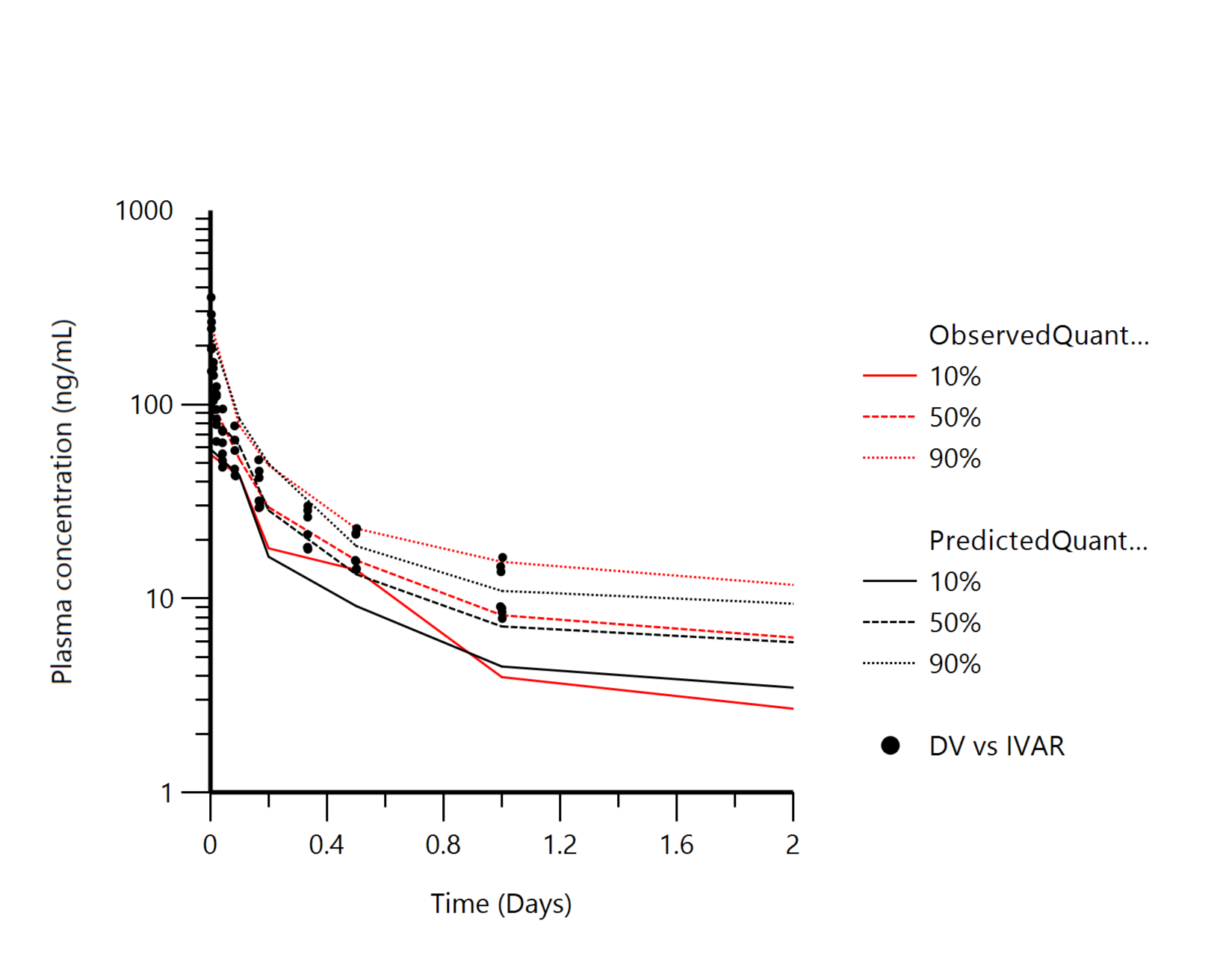
**

**B: obese dogs
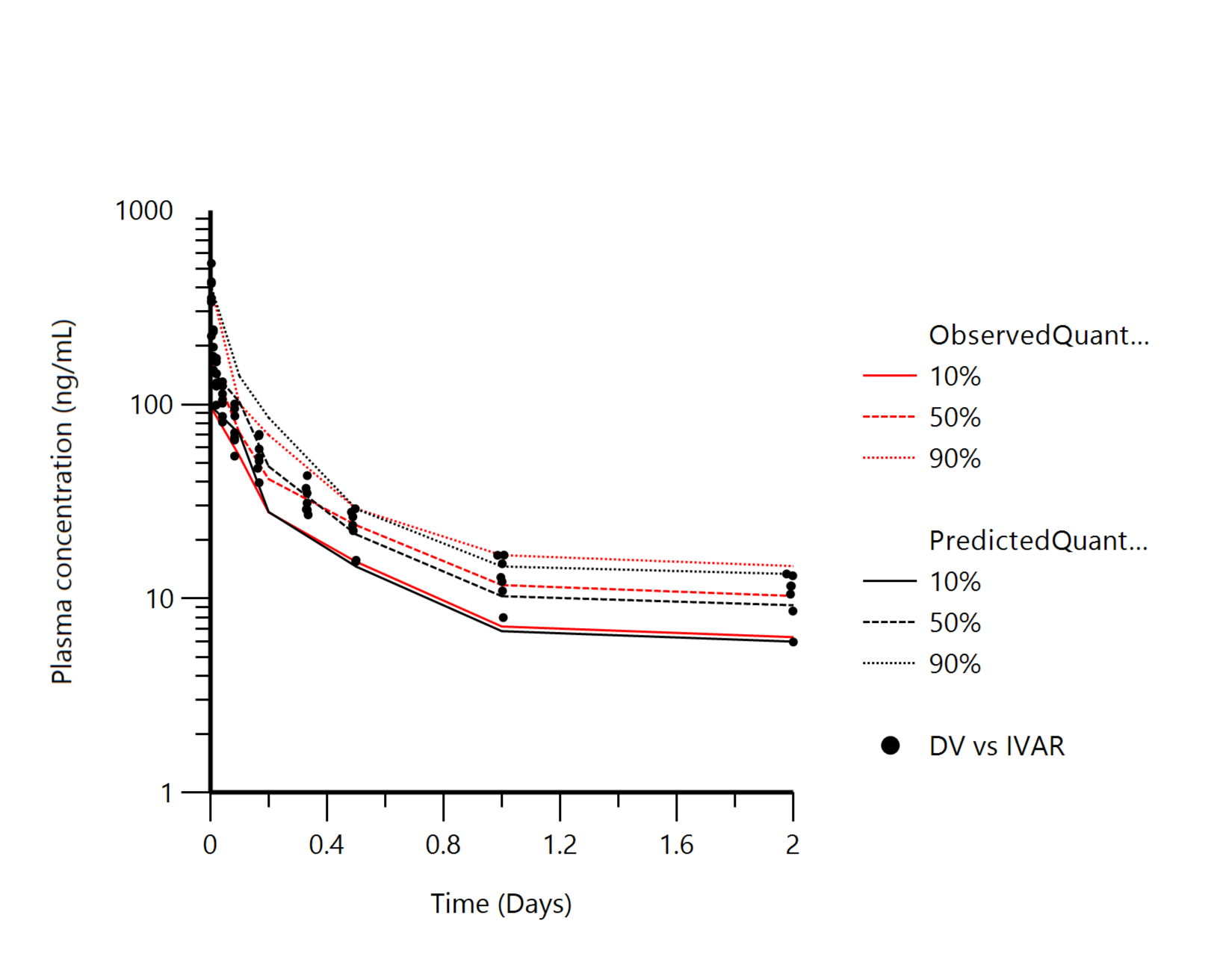
**

**Supplementary figure S4, S5 and S6: individual fitting**

Plot of the dependent variable (DV) i.e. observed plasma concentration (ng/mL) , and individual predicted estimates (IPRED) versus time (Days) latticed by status (lean=blue line vs. obese=red line).

**Supplementary figure S4: Individual fitting for Ivermectin**

**
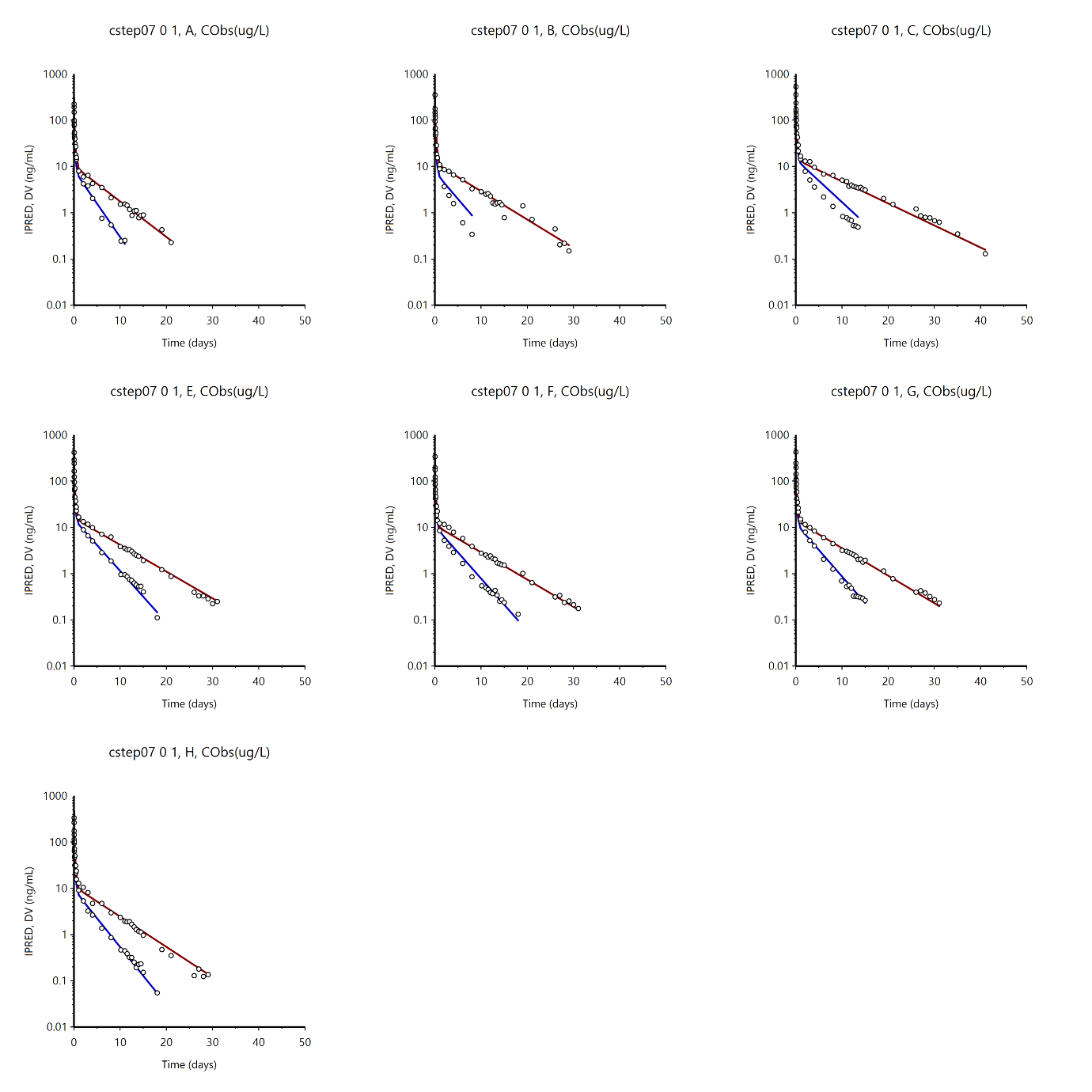
**

**Supplementary figure S5: Individual fitting for Moxidectin**

**
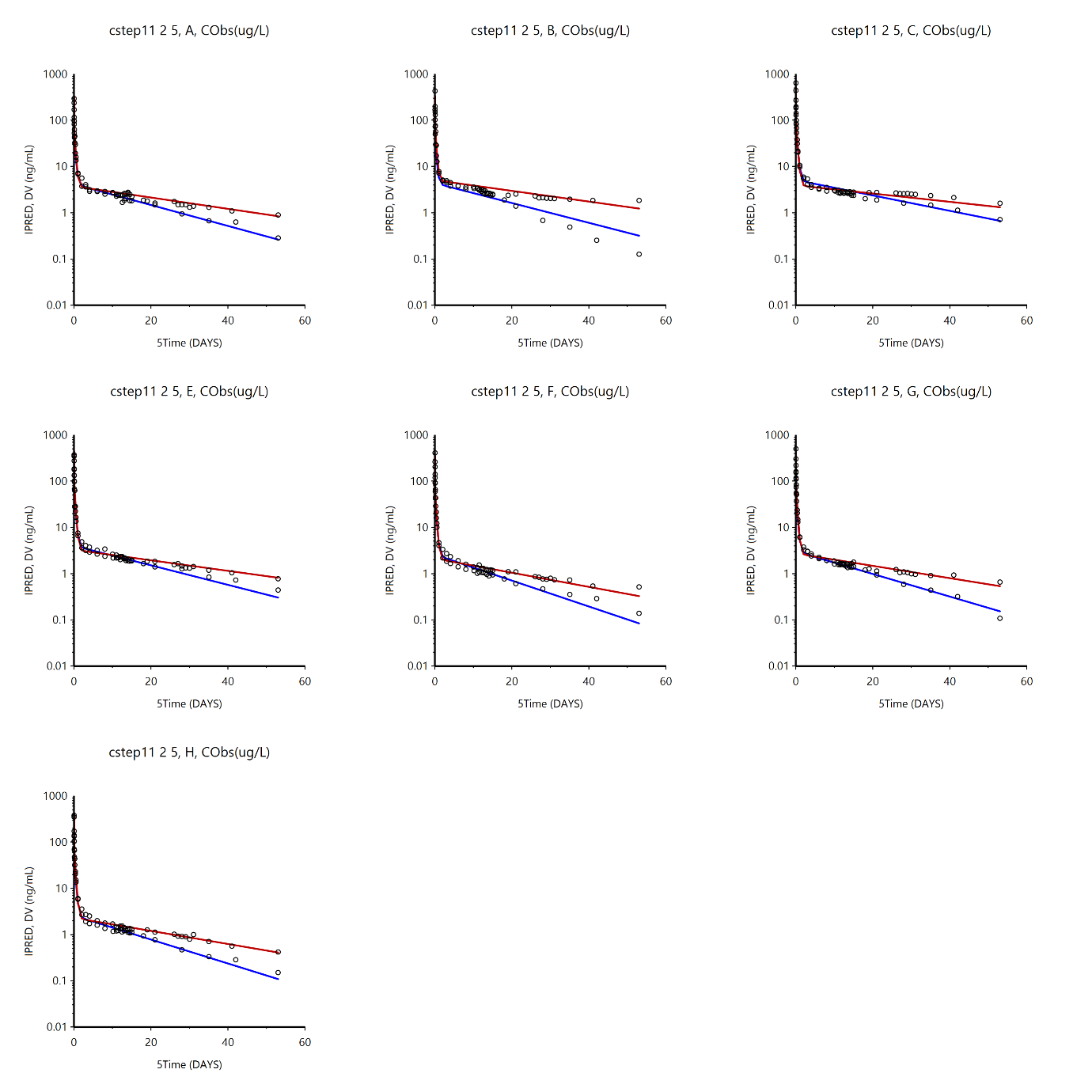
**

**Supplementary figure S6: Individual fitting for Eprinomectin**

**
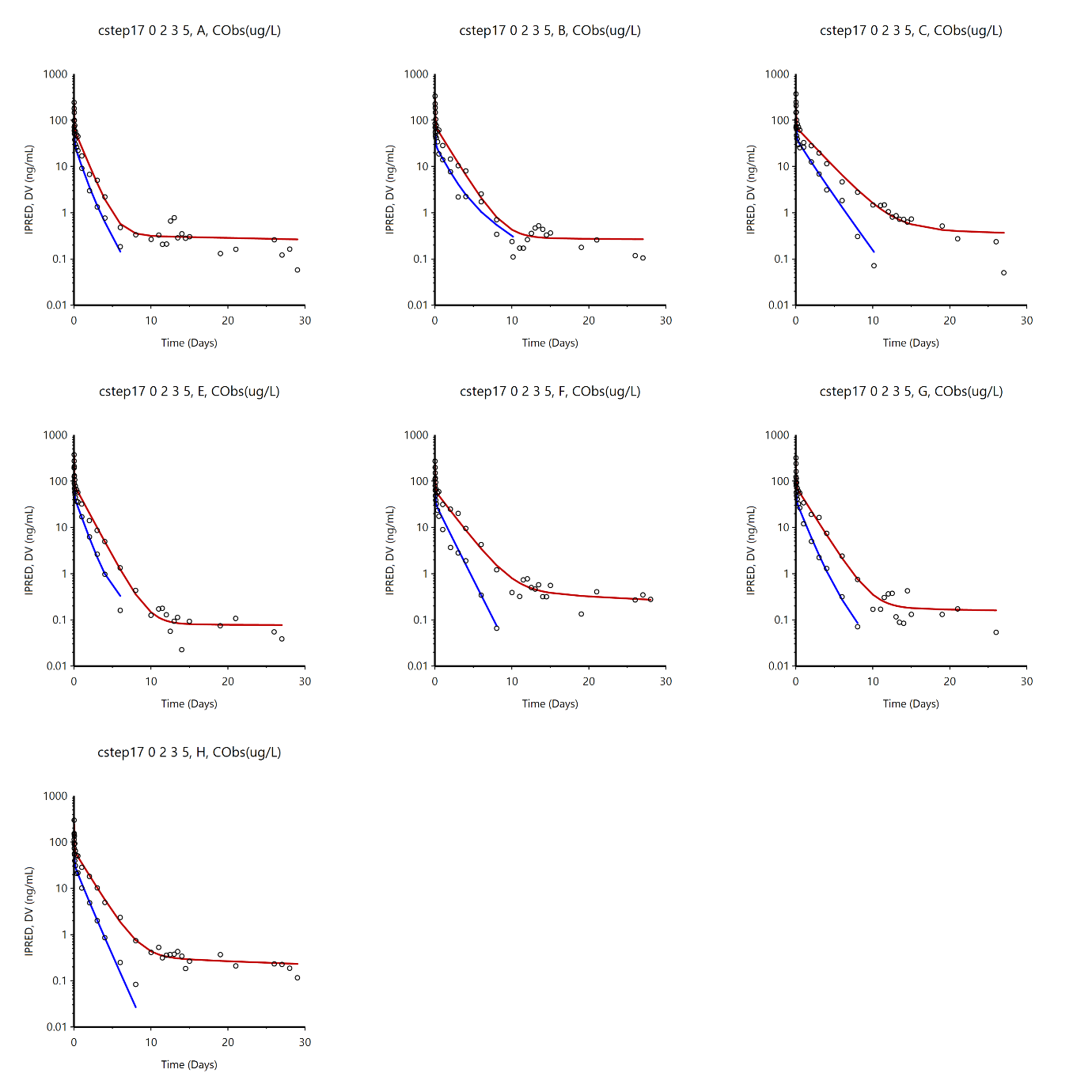
**

**Supplementary Figure S7: Plot of Conditional Weighted Residual values vs. Time for Ivermectin (A), Moxidectin (B) and Eprinomectin (C)**

Plot of CWRES (conditional weighted residuals), a proposed replacement for the classical WRES (weighted residuals) goodness of fit statistic, against IVAR (time). Values of CWRES should be approximately N(0,1) and hence concentrated between y=-2 and y=+2. Ideally, the blue line (average residuals) should be at 0 and the red line (average of positive residuals) with its negative reflection should not show fanning.

**A: Ivermectin**

**
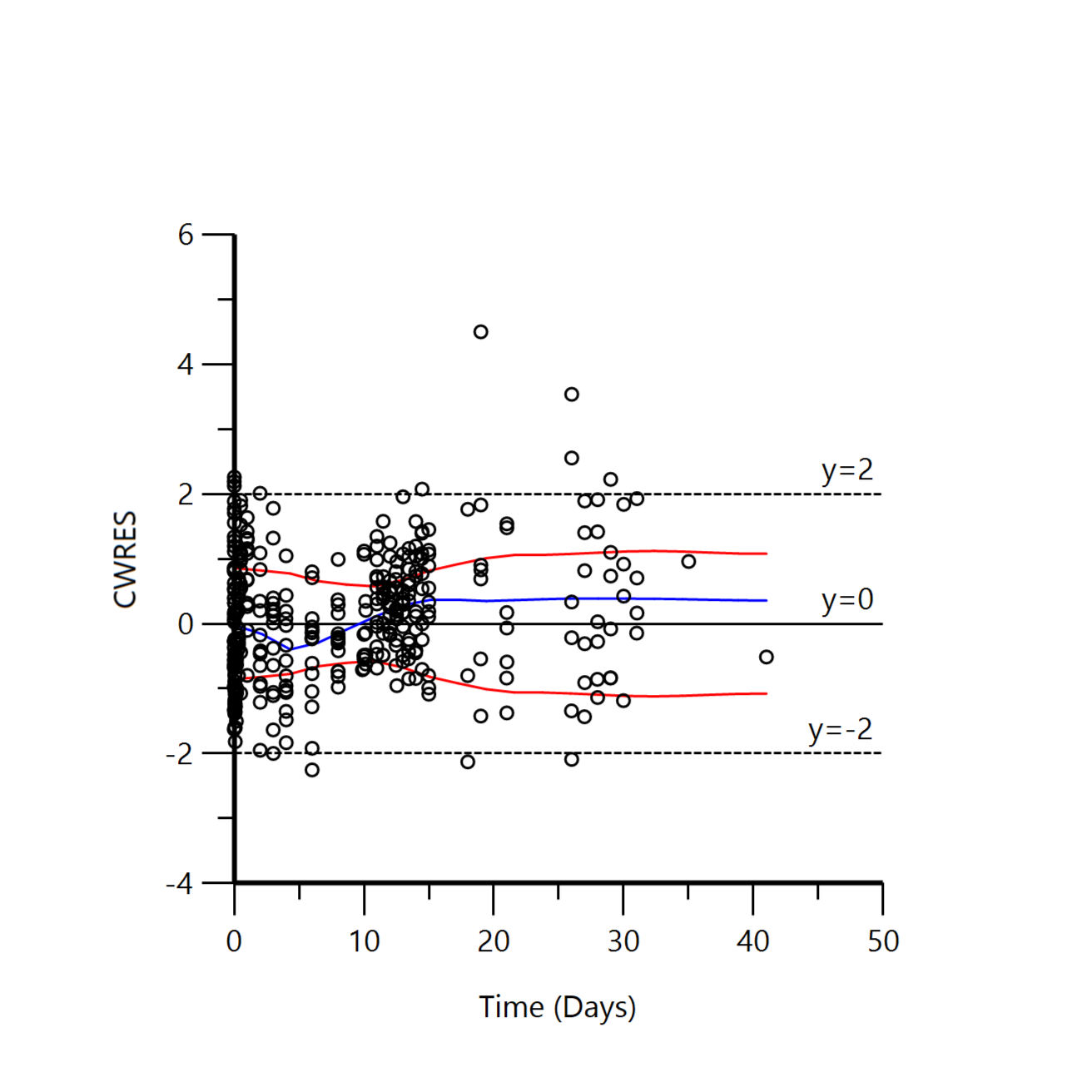
**

**B: Moxidectin**

**
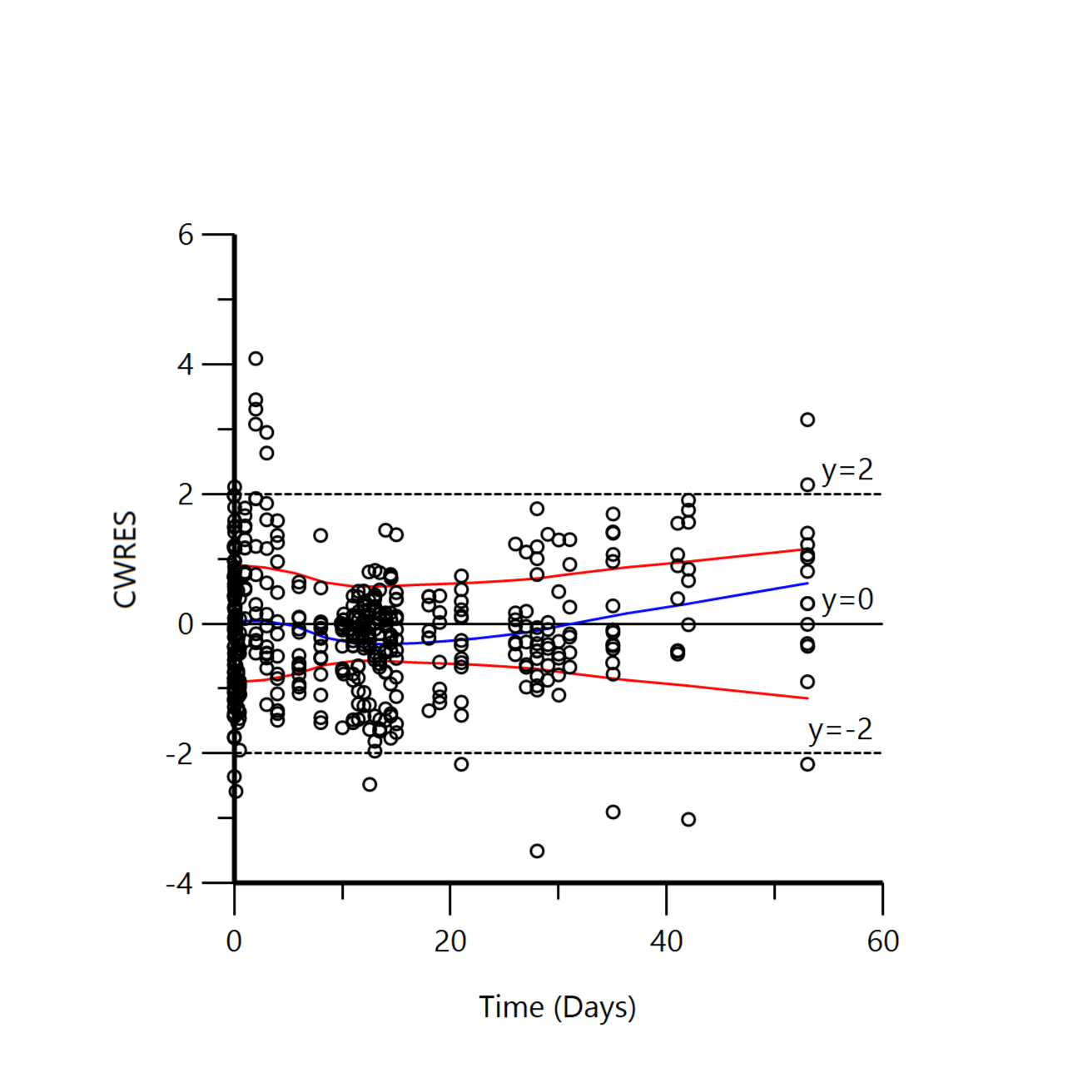
**

**C: Eprinomectin**

**
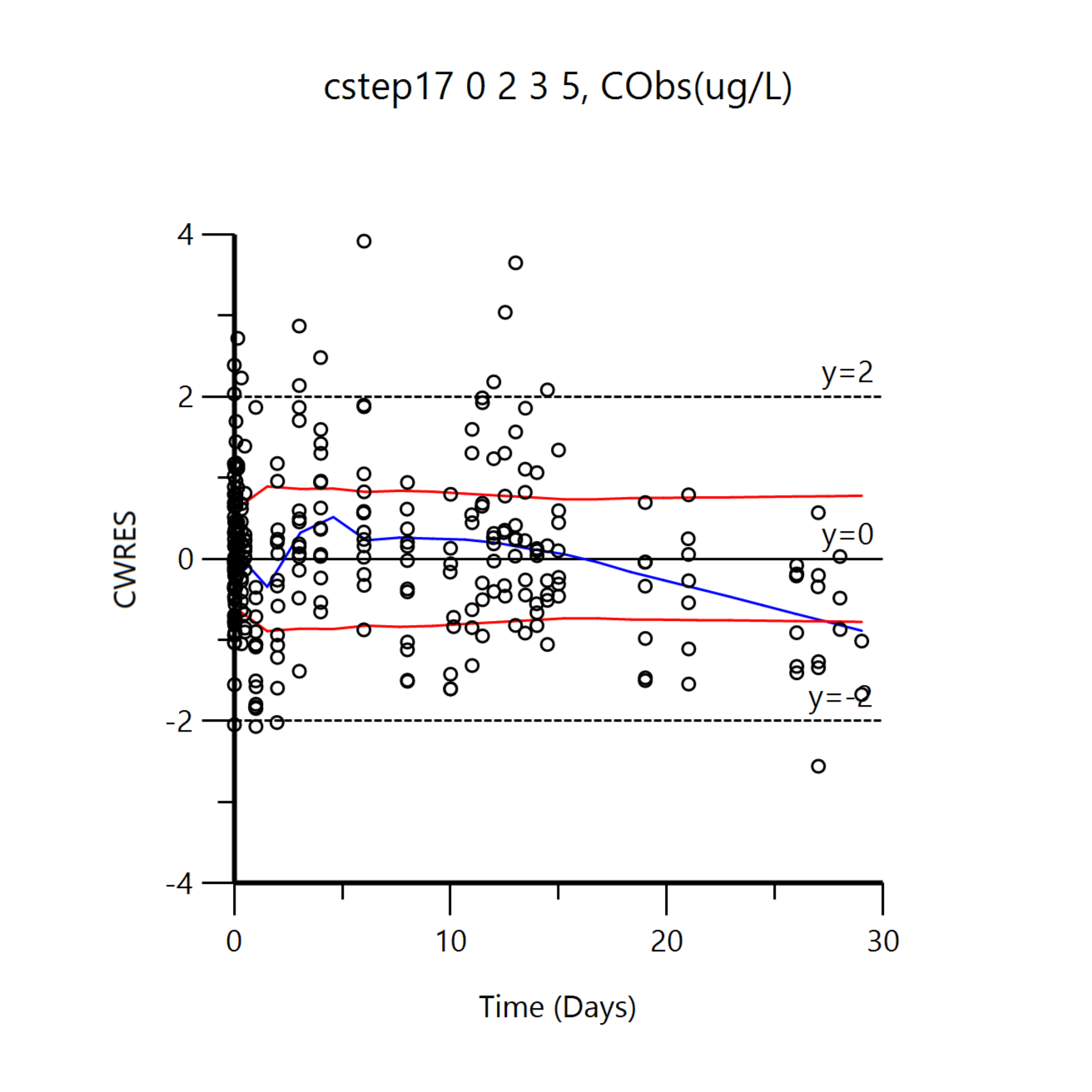
**
