# Supplementary material for: A Large Impact of Obesity on the Disposition of Ivermectin, Moxidectin and Eprinomectin in a Canine Model: Relevance for COVID-19 Patients
Source: Front Pharmacol. 2021 May 20;12:666348. doi: 10.3389/fphar.2021.666348 (PMC8173197; doi:10.3389/fphar.2021.666348)
Supplement: Supplementary file 2 [file datasheet2.docx]

**Supplementary material**

**Supplementary file 2**

# **A** l**arge impact of obesity on the disposition of ivermectin, moxidectin and eprinomectin in a canine model: relevance for COVID-19 patients**

**Running title:** obesity and pharmacokinetics of ivermectin, moxidectin and eprinomectin

**Authors:**

A. Bousquet-Mélou^1^, A Lespine^1^, J-F Sutra^1^, I Bargues^1^, P-L. Toutain^1,3^

^1^ *INTHERES, Université de Toulouse, INRAE, ENVT, Toulouse, France.*

^3^ *The Royal Veterinary College, Hawkshead Campus, Hatfield, Herts., AL9 7TA, United Kingdom*

**Orcid numbers**

Bousquet-Melou***:*** https://orcid.org/0000-0002-7661-4311

P-L Toutain: http://orcid.org/0000-0002-8846-8892

***Corresponding author*: Pierre-Louis Toutain**

**Supplementary Table S4: bootstrap results**

Bootstrap was used as a diagnostic tool to estimate the precision of estimates (Stderror and CV%). The number of samples was n=50; samples were stratified by status (lean vs obese) to ensure that the number of lean and obese dogs were sampled equally for each Bootstrap run. The maximum number of tries (max tries) option was n=5. If a sample failed to converge, it was re-tried up to 5 times, as indicated with different random seeds, in an effort to obtain for evaluation a full set of samples. For EPR, lower and upper bounds were used to prevent spurious estimates from some bootstrap samples, and results (especially precision of estimates) should be interpreted with caution.

Table 4 indicates the mean, standard error, CV%, median and 2.5% and 97.5% percentiles, Status1=obese

| Drugs | Parameter | Mean | Stderr | CV% | Median | 2.50% | 97.50% |
| --- | --- | --- | --- | --- | --- | --- | --- |
| IVM | tvVc | 0.170275 | 0.040101 | 23.55 | 0.166955 | 0.12595 | 0.245831 |
| IVM | tvV2 | 4.589373 | 0.248931 | 5.42 | 4.549732 | 4.214569 | 5.009432 |
| IVM | tvV3 | 26.35412 | 1.549899 | 5.88 | 26.21373 | 23.8218 | 29.13501 |
| IVM | tvClearance | 12.10262 | 0.934493 | 7.72 | 11.94733 | 10.49177 | 13.74773 |
| IVM | tvCld2 | 125.1562 | 16.6434 | 13.30 | 124.2594 | 103.7696 | 157.4394 |
| IVM | tvCld3 | 21.31393 | 0.624353 | 2.93 | 21.27065 | 20.44674 | 22.34432 |
| IVM | tvCMultStdev | 0.207743 | 0.018068 | 8.70 | 0.209377 | 0.243571 | 0.173764 |
| IVM | dVdStatus1 | 0 | 0 | NC | 0 | 0 | 0 |
| IVM | dV2dStatus1 | 0 | 0 | NC | 0 | 0 | 0 |
| IVM | dV3dStatus1 | 0.502348 | 0.082837 | 16.49 | 0.48105 | 0.386875 | 0.667155 |
| IVM | dCldStatus1 | -0.26644 | 0.050049 | 18.78 | -0.26996 | -0.35784 | -0.18421 |
| IVM | dCl2dStatus1 | 0 | 0 | NC | 0 | 0 | 0 |
| IVM | dCl3dStatus1 | 0 | 0 | NC | 0 | 0 | 0 |
| IVM | stdev0 | 0.001 | NC | NC | 0.001 | 0.001 | 0.001 |
|  |  |  |  |  |  |  |  |
| Drug | Parameter | Mean | Stderr | CV% | Median | 2.50% | 97.50% |
| MOXI | tvVc | 2.237063 | 0.168737 | 7.54 | 2.249987 | 1.896609 | 2.507893 |
| MOXI | tvV2 | 6.415554 | 0.311066 | 4.85 | 6.415851 | 5.817608 | 7.005704 |
| MOXI | tvV3 | 104.6299 | 12.26384 | 11.72 | 106.6287 | 74.45826 | 122.7064 |
| MOXI | tvClearance | 7.840601 | 1.016894 | 12.97 | 7.735445 | 6.287474 | 9.554352 |
| MOXI | tvCld2 | 115.9517 | 9.036733 | 7.79 | 114.2763 | 100.5282 | 134.6776 |
| MOXI | tvCld3 | 22.71907 | 1.659005 | 7.30 | 22.59812 | 19.99217 | 26.10045 |
| MOXI | tvCMultStdev | 0.165104 | 0.006148 | 3.72 | 0.166461 | 0.173967 | 0.149426 |
| MOXI | dVdStatus1 | 0 | 0 | NC | 0 | 0 | 0 |
| MOXI | dV2dStatus1 | 0 | 0 | NC | 0 | 0 | 0 |
| MOXI | dV3dStatus1 | 0.683448 | 0.154807 | 22.65 | 0.661784 | 0.461787 | 1.074807 |
| MOXI | dCldStatus1 | 0 | 0 | NC | 0 | 0 | 0 |
| MOXI | dCl2dStatus1 | 0 | 0 | NC | 0 | 0 | 0 |
| MOXI | dCl3dStatus1 | 0.161112 | 0.044431 | 27.58 | 0.161571 | 0.070511 | 0.240966 |
| MOXI | stdev0 | 0.064921 | 0.03604 | 55.51 | 0.065058 | 0.00327 | 0.126139 |
|  |  |  |  |  |  |  |  |
| Drug | Parameter | Mean | Stderr | CV% | Median | 2.50% | 97.50% |
| EPR | tvVc | 6.545981 | 0.394892 | 6.03 | 6.631822 | 5.701018 | 7.083648 |
| EPR | tvV2 | 10.39714 | 0.298367 | 2.87 | 10.39286 | 9.903904 | 11.01787 |
| EPR | tvV3 | 1.357652 | 0.373972 | 27.55 | 1.355979 | 0.708438 | 2.045302 |
| EPR | tvClearance | 14.89758 | 1.108083 | 7.44 | 14.95794 | 12.29464 | 16.71156 |
| EPR | tvCld2 | 89.92221 | 6.610036 | 7.35 | 88.48858 | 79.6952 | 103.0262 |
| EPR | tvCld3 | 0.792946 | 0.176949 | 22.32 | 0.798498 | 0.481177 | 1.089276 |
| EPR | tvCMultStdev | 0.1854 | 0.017402 | 9.39 | 0.18583 | 0.22155 | 0.15616 |
| EPR | dVdStatus1 | -0.57227 | 0.063361 | -11.07 | -0.56754 | -0.7076 | -0.46551 |
| EPR | dV2dStatus1 | 0 | 0 | NC | 0 | 0 | 0 |
| EPR | dV3dStatus1 | 5.025914 | 0.521248 | 10.37 | 5.065701 | 4.034531 | 5.795987 |
| EPR | dCldStatus1 | -0.79114 | 0.122088 | -15.43 | -0.79818 | -0.98348 | -0.54142 |
| EPR | dCl2dStatus1 | 0 | 0 | NC | 0 | 0 | 0 |
| EPR | dCl3dStatus1 | 0.943247 | 0.014534 | 1.54 | 0.942089 | 0.917523 | 0.971519 |
| EPR | stdev0 | 0.108062 | 0.020824 | 19.27 | 0.110923 | 0.064394 | 0.134855 |

*Tv: typical values; Vc: volume of the central compartment; V2: volume of the shallow peripheral compartment; V3: volume of the deep peripheral compartment, Clearance: plasma clearance; Cld2 and Cld3: distribution clearance for the shallow and deep compartment ; tvCMultStdev: multiplicative component of the error model is expressed as CV% and the additive component of the residual error model by its standard deviation.) dVdStatus1 are the estimate of the fixed effect for covariates (exponential model).*

**Supplementary table S5: Omega matrix and ETA shrinkage for Ivermectin, Moxidectin and Eprinomectin**

Estimates of the random effects variance-covariance matrix, correlation matrix and shrinkage.

These matrices were computed in a single run. Diagonals are variance terms and off diagonals are covariance terms.

| Drugs | Label | nVc | nClearance | nV2 | nCld2 | nV3 | nCld3 |
| --- | --- | --- | --- | --- | --- | --- | --- |
| IVM | **Omega** |  |  |  |  |  |  |
| IVM | nVc | 0.015598 |  |  |  |  |  |
| IVM | nClearance | -0.00208 | 0.038349099 |  |  |  |  |
| IVM | nV2 | 0.000433 | 0.013226495 | 0.011163 |  |  |  |
| IVM | nCld2 | 0.006196 | -0.003480704 | 0.0013 | 0.011272 |  |  |
| IVM | nV3 | -0.00177 | 0.005685147 | 0.003358 | -0.00092 | 0.006781 |  |
| IVM | nCld3 | -0.00075 | 0.005200032 | 0.001289 | 0.000595 | 0.002017 | 0.007352 |
| IVM | **Correlation** |  |  |  |  |  |  |
| IVM | nVc | 1 |  |  |  |  |  |
| IVM | nClearance | -0.08501 | 1 |  |  |  |  |
| IVM | nV2 | 0.032827 | 0.639246821 | 1 |  |  |  |
| IVM | nCld2 | 0.46731 | -0.16741505 | 0.11589 | 1 |  |  |
| IVM | nV3 | -0.17186 | 0.352534436 | 0.385924 | -0.10479 | 1 |  |
| IVM | nCld3 | -0.06961 | 0.309685493 | 0.142265 | 0.065323 | 0.285593 | 1 |
| IVM | **Shrinkage** | **0.48** | **-0.07** | **0.20** | **0.72** | **0.18** | **0.41** |
|  |  |  |  |  |  |  |  |
| Scenario | Label | nVc | nClearance | nV2 | nCld2 | nV3 | nCld3 |
| MOX | **Omega** |  |  |  |  |  |  |
| MOX | nVc | 0.061259 |  |  |  |  |  |
| MOX | nClearance | -0.003 | 0.088848894 |  |  |  |  |
| MOX | nV2 | 0.016874 | 0.013124321 | 0.009248 |  |  |  |
| MOX | nCld2 | 0.000231 | -0.000137913 | 6.2E-05 | 1.21E-06 |  |  |
| MOX | nV3 | -0.00541 | 0.022833568 | 0.00154 | -5.7E-05 | 0.007495 |  |
| MOX | nCld3 | 0.040999 | -0.00212481 | 0.011411 | 0.000156 | -0.00397 | 0.027517 |
| MOX | **Correlation** |  |  |  |  |  |  |
| MOX | nVc | 1 |  |  |  |  |  |
| MOX | nClearance | -0.04064 | 1 |  |  |  |  |
| MOX | nV2 | 0.708956 | 0.457860755 | 1 |  |  |  |
| MOX | nCld2 | 0.848053 | -0.41989515 | 0.585143 | 1 |  |  |
| MOX | nV3 | -0.25262 | 0.884861057 | 0.184988 | -0.593 | 1 |  |
| MOX | nCld3 | 0.998586 | -0.042972408 | 0.715296 | 0.853396 | -0.27624 | 1 |
| MOX | **Shrinkage** | **0.05** | **0.01** | **0.12** | **0.08** | **0.06** | **0.04** |
|  |  |  |  |  |  |  |  |
| Scenario | Label | nVc | nClearance | nV2 | nCld2 | nV3 | nCld3 |
| EPR | **Omega** |  |  |  |  |  |  |
| EPR | nVc | 0.029681 |  |  |  |  |  |
| EPR | nClearance | 0.015945 | 0.056048071 |  |  |  |  |
| EPR | nV2 | 0.007734 | -0.000203799 | 0.002447 |  |  |  |
| EPR | nCld2 | -0.00504 | 0.00835591 | -0.00237 | 0.003491 |  |  |
| EPR | nV3 | -0.08949 | -0.077735667 | -0.02195 | 0.010046 | 1.209932 |  |
| EPR | nCld3 | 0.027782 | 0.024465504 | 0.006315 | -0.00243 | 0.359834 | 0.260515 |
| EPR | **Correlation** |  |  |  |  |  |  |
| EPR | nVc | 1 |  |  |  |  |  |
| EPR | nClearance | 0.390946 | 1 |  |  |  |  |
| EPR | nV2 | 0.907479 | -0.017402669 | 1 |  |  |  |
| EPR | nCld2 | -0.49473 | 0.597355689 | -0.81103 | 1 |  |  |
| EPR | nV3 | -0.47224 | -0.298510383 | -0.40349 | 0.154578 | 1 |  |
| EPR | nCld3 | 0.315943 | 0.202468391 | 0.25014 | -0.08053 | 0.640922 | 1 |
| EPR | **Shrinkage** | **0.21** | **0.09** | **0.13** | **0.01** | **0.01** | **-0.09** |
