# Supplementary material for: A Large Impact of Obesity on the Disposition of Ivermectin, Moxidectin and Eprinomectin in a Canine Model: Relevance for COVID-19 Patients
Source: Front Pharmacol. 2021 May 20;12:666348. doi: 10.3389/fphar.2021.666348 (PMC8173197; doi:10.3389/fphar.2021.666348)
Supplement: Supplementary file 3 [file datasheet1.docx]

**Supplementary material**

**Supplementary file 1**

# **A** l**arge impact of obesity on the disposition of ivermectin, moxidectin and eprinomectin in a canine model: relevance for COVID-19 patients**

**Running title:** obesity and pharmacokinetics of ivermectin, moxidectin and eprinomectin

**Authors:**

A. Bousquet-Mélou^1^, A Lespine^1^, J-F Sutra^1^, I Bargues^1^, P-L. Toutain^1,3^

^1^ *INTHERES, Université de Toulouse, INRAE, ENVT, Toulouse, France.*

^3^ *The Royal Veterinary College, Hawkshead Campus, Hatfield, Herts., AL9 7TA, United Kingdom*

**Orcid numbers**

Bousquet-Melou***:*** https://orcid.org/0000-0002-7661-4311

P-L Toutain: http://orcid.org/0000-0002-8846-8892

***Corresponding author*: Pierre-Louis Toutain**

**Supplementary Table S1: Ivermectin raw data**

| Status | Dogs | Time | Cobs | dose_total | Body Weight | %Body Fat |
| --- | --- | --- | --- | --- | --- | --- |
|  | Units | day | ug/L | ug in toto | Kg |  |
| Lean | A | 0 |  | 694 | 10.3 | 20.58 |
| Lean | A | 0.003472 | 192.5171 |  |  |  |
| Lean | A | 0.011111 | 93.93818 |  |  |  |
| Lean | A | 0.020833 | 78.63112 |  |  |  |
| Lean | A | 0.041667 | 51.27303 |  |  |  |
| Lean | A | 0.084722 | 43.15917 |  |  |  |
| Lean | A | 0.170139 | 31.19796 |  |  |  |
| Lean | A | 0.334689 | 17.96614 |  |  |  |
| Lean | A | 0.501389 | 14.2554 |  |  |  |
| Lean | A | 1.001389 | 7.900538 |  |  |  |
| Lean | A | 2.00625 | 4.217862 |  |  |  |
| Lean | A | 3.006944 | 3.836121 |  |  |  |
| Lean | A | 4.004861 | 2.039694 |  |  |  |
| Lean | A | 6.002778 | 0.755997 |  |  |  |
| Lean | A | 8.013194 | 0.542671 |  |  |  |
| Lean | A | 10.14583 | 0.243266 |  |  |  |
| Lean | A | 11 | 0.250751 |  |  |  |
| Obese | A | 0 |  | 1136 | 16.5 | 34.53 |
| Obese | A | 0.003467 | 224.8127 |  |  |  |
| Obese | A | 0.0104 | 150.215 |  |  |  |
| Obese | A | 0.020106 | 99.23336 |  |  |  |
| Obese | A | 0.0417 | 81.02996 |  |  |  |
| Obese | A | 0.08335 | 54.14741 |  |  |  |
| Obese | A | 0.167394 | 39.50554 |  |  |  |
| Obese | A | 0.335383 | 26.99993 |  |  |  |
| Obese | A | 0.5 | 15.75527 |  |  |  |
| Obese | A | 1.004861 | 7.981595 |  |  |  |
| Obese | A | 2 | 5.977813 |  |  |  |
| Obese | A | 3.006944 | 6.428873 |  |  |  |
| Obese | A | 4.002083 | 4.289269 |  |  |  |
| Obese | A | 6.004861 | 3.511231 |  |  |  |
| Obese | A | 8.010417 | 2.106068 |  |  |  |
| Obese | A | 10.00556 | 1.529247 |  |  |  |
| Obese | A | 10.99653 | 1.546015 |  |  |  |
| Obese | A | 11.47778 | 1.44876 |  |  |  |
| Obese | A | 12.00694 | 1.173764 |  |  |  |
| Obese | A | 12.52778 | 0.873987 |  |  |  |
| Obese | A | 13.01042 | 1.089872 |  |  |  |
| Obese | A | 13.46181 | 1.107282 |  |  |  |
| Obese | A | 13.99792 | 0.778231 |  |  |  |
| Obese | A | 14.47292 | 0.877469 |  |  |  |
| Obese | A | 14.99792 | 0.891397 |  |  |  |
| Obese | A | 19.00486 | 0.428288 |  |  |  |
| Obese | A | 21.00903 | 0.226331 |  |  |  |
| Lean | B | 0 |  | 600 | 9.25 | 15.85 |
| Lean | B | 0.003467 | 148.1581 |  |  |  |
| Lean | B | 0.0104 | 94.24484 |  |  |  |
| Lean | B | 0.0208 | 64.46385 |  |  |  |
| Lean | B | 0.0417 | 47.45961 |  |  |  |
| Lean | B | 0.1667 | 29.34592 |  |  |  |
| Lean | B | 0.3333 | 18.32224 |  |  |  |
| Lean | B | 0.499306 | 15.58299 |  |  |  |
| Lean | B | 1 | 8.911819 |  |  |  |
| Lean | B | 2.003472 | 3.661895 |  |  |  |
| Lean | B | 3.011111 | 2.363916 |  |  |  |
| Lean | B | 4.004167 | 1.578965 |  |  |  |
| Lean | B | 6.002083 | 0.609107 |  |  |  |
| Lean | B | 8.011111 | 0.34081 |  |  |  |
| Obese | B | 0 |  | 1277 | 18.8 | 40.96 |
| Obese | B | 0.003467 | 352.5138 |  |  |  |
| Obese | B | 0.009706 | 176.9156 |  |  |  |
| Obese | B | 0.0208 | 129.0209 |  |  |  |
| Obese | B | 0.0417 | 113.3432 |  |  |  |
| Obese | B | 0.08335 | 67.82694 |  |  |  |
| Obese | B | 0.1667 | 53.34345 |  |  |  |
| Obese | B | 0.332606 | 28.62104 |  |  |  |
| Obese | B | 0.498611 | 15.47479 |  |  |  |
| Obese | B | 1.002083 | 10.94745 |  |  |  |
| Obese | B | 1.998611 | 8.623901 |  |  |  |
| Obese | B | 3.009722 | 7.853821 |  |  |  |
| Obese | B | 3.999306 | 6.563147 |  |  |  |
| Obese | B | 6.002083 | 5.172678 |  |  |  |
| Obese | B | 8.009028 | 3.306522 |  |  |  |
| Obese | B | 10.00347 | 2.850794 |  |  |  |
| Obese | B | 10.99653 | 2.50983 |  |  |  |
| Obese | B | 11.47569 | 2.588002 |  |  |  |
| Obese | B | 12.00347 | 2.291945 |  |  |  |
| Obese | B | 12.51944 | 1.626119 |  |  |  |
| Obese | B | 13.00833 | 1.5392 |  |  |  |
| Obese | B | 13.46042 | 1.629741 |  |  |  |
| Obese | B | 13.99444 | 1.658714 |  |  |  |
| Obese | B | 14.47431 | 1.477632 |  |  |  |
| Obese | B | 14.99514 | 0.782276 |  |  |  |
| Obese | B | 19.00278 | 1.408821 |  |  |  |
| Obese | B | 21.00694 | 0.717086 |  |  |  |
| Obese | B | 26.00208 | 0.445463 |  |  |  |
| Obese | B | 27.00347 | 0.202812 |  |  |  |
| Obese | B | 28.00347 | 0.217299 |  |  |  |
| Obese | B | 29.00347 | 0.148488 |  |  |  |
| Lean | C | 0 |  | 803 | 11.6 | 28.405 |
| Lean | C | 0.002772 | 355.8548 |  |  |  |
| Lean | C | 0.009706 | 153.4748 |  |  |  |
| Lean | C | 0.0208 | 112.7312 |  |  |  |
| Lean | C | 0.041006 | 73.38461 |  |  |  |
| Lean | C | 0.08335 | 77.52849 |  |  |  |
| Lean | C | 0.166006 | 51.80348 |  |  |  |
| Lean | C | 0.334689 | 29.99344 |  |  |  |
| Lean | C | 0.499306 | 21.70569 |  |  |  |
| Lean | C | 0.995833 | 14.61296 |  |  |  |
| Lean | C | 2.001389 | 7.821521 |  |  |  |
| Lean | C | 3.009028 | 5.099894 |  |  |  |
| Lean | C | 3.999306 | 3.585074 |  |  |  |
| Lean | C | 5.999306 | 2.194806 |  |  |  |
| Lean | C | 8.00625 | 1.366704 |  |  |  |
| Lean | C | 10.1375 | 0.828102 |  |  |  |
| Lean | C | 10.99167 | 0.777608 |  |  |  |
| Lean | C | 11.50278 | 0.715332 |  |  |  |
| Lean | C | 11.99375 | 0.679986 |  |  |  |
| Lean | C | 12.45972 | 0.525138 |  |  |  |
| Lean | C | 12.98681 | 0.515039 |  |  |  |
| Lean | C | 13.45 | 0.488109 |  |  |  |
| Obese | C | 0 |  | 1126 | 16.6 | 40.03 |
| Obese | C | 0.003467 | 532.3462 |  |  |  |
| Obese | C | 0.0104 | 236.8697 |  |  |  |
| Obese | C | 0.0208 | 172.6948 |  |  |  |
| Obese | C | 0.0417 | 130.8027 |  |  |  |
| Obese | C | 0.08335 | 100.4644 |  |  |  |
| Obese | C | 0.1667 | 69.04962 |  |  |  |
| Obese | C | 0.332606 | 42.98223 |  |  |  |
| Obese | C | 0.497222 | 29.01706 |  |  |  |
| Obese | C | 1.007639 | 16.74233 |  |  |  |
| Obese | C | 1.997917 | 13.11305 |  |  |  |
| Obese | C | 3.001389 | 12.77496 |  |  |  |
| Obese | C | 4.001389 | 9.632103 |  |  |  |
| Obese | C | 6.001389 | 6.865289 |  |  |  |
| Obese | C | 8.008333 | 6.430602 |  |  |  |
| Obese | C | 10.00278 | 5.078244 |  |  |  |
| Obese | C | 10.99583 | 4.755679 |  |  |  |
| Obese | C | 11.47639 | 3.738172 |  |  |  |
| Obese | C | 12.00278 | 3.975709 |  |  |  |
| Obese | C | 12.52292 | 3.682284 |  |  |  |
| Obese | C | 13.00764 | 3.569598 |  |  |  |
| Obese | C | 13.45903 | 3.453277 |  |  |  |
| Obese | C | 13.99722 | 3.525978 |  |  |  |
| Obese | C | 14.47708 | 3.255168 |  |  |  |
| Obese | C | 14.99514 | 3.097044 |  |  |  |
| Obese | C | 19.00278 | 2.031981 |  |  |  |
| Obese | C | 21.00625 | 1.523077 |  |  |  |
| Obese | C | 26.00278 | 1.205275 |  |  |  |
| Obese | C | 27.00278 | 0.857742 |  |  |  |
| Obese | C | 28.00208 | 0.793041 |  |  |  |
| Obese | C | 29.00278 | 0.772707 |  |  |  |
| Obese | C | 30.00556 | 0.669186 |  |  |  |
| Obese | C | 31.02361 | 0.624821 |  |  |  |
| Obese | C | 35.00972 | 0.347533 |  |  |  |
| Obese | C | 41.00139 | 0.129401 |  |  |  |
| Lean | E | 0 |  | 777 | 11.3 | 27.315 |
| Lean | E | 0.003467 | 290.8455 |  |  |  |
| Lean | E | 0.0104 | 164.7317 |  |  |  |
| Lean | E | 0.0208 | 123.5204 |  |  |  |
| Lean | E | 0.042394 | 94.71617 |  |  |  |
| Lean | E | 0.084044 | 65.576 |  |  |  |
| Lean | E | 0.167394 | 45.32151 |  |  |  |
| Lean | E | 0.333994 | 28.44331 |  |  |  |
| Lean | E | 0.502083 | 22.96338 |  |  |  |
| Lean | E | 1.002778 | 16.29585 |  |  |  |
| Lean | E | 2.000694 | 8.902031 |  |  |  |
| Lean | E | 3.005556 | 6.579193 |  |  |  |
| Lean | E | 3.995139 | 5.1037 |  |  |  |
| Lean | E | 5.994444 | 2.856109 |  |  |  |
| Lean | E | 8.003472 | 1.881171 |  |  |  |
| Lean | E | 10.13056 | 0.955308 |  |  |  |
| Lean | E | 10.98542 | 0.945493 |  |  |  |
| Lean | E | 11.49583 | 0.860431 |  |  |  |
| Lean | E | 11.9875 | 0.752469 |  |  |  |
| Lean | E | 12.45556 | 0.713209 |  |  |  |
| Lean | E | 13.00694 | 0.628148 |  |  |  |
| Lean | E | 13.44792 | 0.565987 |  |  |  |
| Lean | E | 13.99375 | 0.526728 |  |  |  |
| Lean | E | 14.45556 | 0.533271 |  |  |  |
| Lean | E | 14.99167 | 0.404043 |  |  |  |
| Lean | E | 17.99722 | 0.111234 |  |  |  |
| Obese | E | 0 |  | 1150 | 16.8 | 40.8 |
| Obese | E | 0.003467 | 419.4309 |  |  |  |
| Obese | E | 0.0104 | 242.3557 |  |  |  |
| Obese | E | 0.0208 | 165.6834 |  |  |  |
| Obese | E | 0.0417 | 124.3876 |  |  |  |
| Obese | E | 0.08335 | 95.18633 |  |  |  |
| Obese | E | 0.167394 | 70.09227 |  |  |  |
| Obese | E | 0.328439 | 36.9785 |  |  |  |
| Obese | E | 0.484028 | 27.87804 |  |  |  |
| Obese | E | 0.985417 | 16.71155 |  |  |  |
| Obese | E | 1.977083 | 13.3891 |  |  |  |
| Obese | E | 2.990278 | 11.73169 |  |  |  |
| Obese | E | 3.982639 | 9.871882 |  |  |  |
| Obese | E | 5.98125 | 7.14518 |  |  |  |
| Obese | E | 7.988194 | 6.224823 |  |  |  |
| Obese | E | 9.981944 | 3.85328 |  |  |  |
| Obese | E | 10.97778 | 3.547767 |  |  |  |
| Obese | E | 11.45556 | 3.31318 |  |  |  |
| Obese | E | 11.98194 | 3.322061 |  |  |  |
| Obese | E | 12.49097 | 3.036999 |  |  |  |
| Obese | E | 12.98681 | 2.700773 |  |  |  |
| Obese | E | 13.4375 | 2.510732 |  |  |  |
| Obese | E | 13.97569 | 2.38282 |  |  |  |
| Obese | E | 14.97639 | 1.925991 |  |  |  |
| Obese | E | 18.98403 | 1.213338 |  |  |  |
| Obese | E | 20.98542 | 0.869803 |  |  |  |
| Obese | E | 25.98333 | 0.3947 |  |  |  |
| Obese | E | 26.98333 | 0.328917 |  |  |  |
| Obese | E | 27.98125 | 0.328917 |  |  |  |
| Obese | E | 28.98194 | 0.285061 |  |  |  |
| Obese | E | 29.98472 | 0.222933 |  |  |  |
| Obese | E | 31.00694 | 0.248515 |  |  |  |
| Lean | F | 0 |  | 689 | 10.3 | 27.475 |
| Lean | F | 0.004161 | 197.0216 |  |  |  |
| Lean | F | 0.0104 | 104.4383 |  |  |  |
| Lean | F | 0.0208 | 84.22179 |  |  |  |
| Lean | F | 0.0417 | 55.69611 |  |  |  |
| Lean | F | 0.086822 | 42.81163 |  |  |  |
| Lean | F | 0.170172 | 29.72062 |  |  |  |
| Lean | F | 0.3333 | 18.37426 |  |  |  |
| Lean | F | 0.5 | 14.06505 |  |  |  |
| Lean | F | 1.000694 | 8.507459 |  |  |  |
| Lean | F | 2.017361 | 5.227772 |  |  |  |
| Lean | F | 3.004167 | 3.899252 |  |  |  |
| Lean | F | 3.993056 | 2.891303 |  |  |  |
| Lean | F | 5.990278 | 1.66142 |  |  |  |
| Lean | F | 8.006944 | 0.856911 |  |  |  |
| Lean | F | 10.12708 | 0.545587 |  |  |  |
| Lean | F | 10.98264 | 0.493186 |  |  |  |
| Lean | F | 11.49306 | 0.454656 |  |  |  |
| Lean | F | 11.98542 | 0.391466 |  |  |  |
| Lean | F | 12.45278 | 0.36989 |  |  |  |
| Lean | F | 13.00486 | 0.43462 |  |  |  |
| Lean | F | 13.44583 | 0.342148 |  |  |  |
| Lean | F | 13.99167 | 0.252758 |  |  |  |
| Lean | F | 14.45625 | 0.271252 |  |  |  |
| Lean | F | 14.98889 | 0.237346 |  |  |  |
| Lean | F | 17.99583 | 0.132544 |  |  |  |
| Obese | F | 0 |  | 1001 | 15.1 | 35.38 |
| Obese | F | 0.003467 | 341.4361 |  |  |  |
| Obese | F | 0.0104 | 175.1879 |  |  |  |
| Obese | F | 0.0208 | 124.1459 |  |  |  |
| Obese | F | 0.0417 | 86.72511 |  |  |  |
| Obese | F | 0.08335 | 65.56441 |  |  |  |
| Obese | F | 0.162533 | 46.88814 |  |  |  |
| Obese | F | 0.329133 | 28.80425 |  |  |  |
| Obese | F | 0.490278 | 22.34906 |  |  |  |
| Obese | F | 1.000694 | 12.22825 |  |  |  |
| Obese | F | 1.993056 | 11.60356 |  |  |  |
| Obese | F | 3.00625 | 9.998734 |  |  |  |
| Obese | F | 3.997917 | 7.86256 |  |  |  |
| Obese | F | 5.996528 | 5.787418 |  |  |  |
| Obese | F | 8.005556 | 3.902558 |  |  |  |
| Obese | F | 9.997222 | 2.775232 |  |  |  |
| Obese | F | 10.99236 | 2.520327 |  |  |  |
| Obese | F | 11.46597 | 2.276193 |  |  |  |
| Obese | F | 11.99653 | 2.416211 |  |  |  |
| Obese | F | 12.50278 | 2.132585 |  |  |  |
| Obese | F | 13.00347 | 2.05001 |  |  |  |
| Obese | F | 13.45208 | 1.70894 |  |  |  |
| Obese | F | 13.99306 | 1.640726 |  |  |  |
| Obese | F | 14.47569 | 1.565331 |  |  |  |
| Obese | F | 14.99167 | 1.518659 |  |  |  |
| Obese | F | 19 | 1.012439 |  |  |  |
| Obese | F | 21.00139 | 0.639057 |  |  |  |
| Obese | F | 25.99931 | 0.312348 |  |  |  |
| Obese | F | 26.99931 | 0.33748 |  |  |  |
| Obese | F | 27.99861 | 0.236954 |  |  |  |
| Obese | F | 29.00347 | 0.254905 |  |  |  |
| Obese | F | 30 | 0.211822 |  |  |  |
| Obese | F | 31.02639 | 0.17592 |  |  |  |
| Lean | G | 0 |  | 705 | 10.6 | 24.365 |
| Lean | G | 0.003467 | 245.422 |  |  |  |
| Lean | G | 0.011094 | 140.9691 |  |  |  |
| Lean | G | 0.0208 | 110.1262 |  |  |  |
| Lean | G | 0.042394 | 72.54109 |  |  |  |
| Lean | G | 0.084044 | 57.91117 |  |  |  |
| Lean | G | 0.167394 | 41.96014 |  |  |  |
| Lean | G | 0.333994 | 26.27918 |  |  |  |
| Lean | G | 0.499306 | 21.42749 |  |  |  |
| Lean | G | 0.997222 | 13.74158 |  |  |  |
| Lean | G | 2.020833 | 7.895794 |  |  |  |
| Lean | G | 3.002778 | 5.206376 |  |  |  |
| Lean | G | 3.990278 | 3.989386 |  |  |  |
| Lean | G | 5.990972 | 2.046757 |  |  |  |
| Lean | G | 8.00625 | 1.24953 |  |  |  |
| Lean | G | 9.876389 | 0.693099 |  |  |  |
| Lean | G | 10.97917 | 0.527146 |  |  |  |
| Lean | G | 11.49028 | 0.558058 |  |  |  |
| Lean | G | 11.98194 | 0.48159 |  |  |  |
| Lean | G | 12.45556 | 0.325398 |  |  |  |
| Lean | G | 13.00208 | 0.322145 |  |  |  |
| Lean | G | 13.44375 | 0.315637 |  |  |  |
| Lean | G | 13.98889 | 0.305875 |  |  |  |
| Lean | G | 14.45417 | 0.292859 |  |  |  |
| Lean | G | 14.98611 | 0.263573 |  |  |  |
| Obese | G | 0 |  | 1125 | 17 | 38.78 |
| Obese | G | 0.003467 | 428.3598 |  |  |  |
| Obese | G | 0.0104 | 197.2017 |  |  |  |
| Obese | G | 0.0208 | 143.7848 |  |  |  |
| Obese | G | 0.0417 | 105.4118 |  |  |  |
| Obese | G | 0.084044 | 87.18371 |  |  |  |
| Obese | G | 0.167394 | 58.96389 |  |  |  |
| Obese | G | 0.331911 | 34.91491 |  |  |  |
| Obese | G | 0.488889 | 26.38366 |  |  |  |
| Obese | G | 1.001389 | 15.11751 |  |  |  |
| Obese | G | 1.990972 | 11.60495 |  |  |  |
| Obese | G | 3.008333 | 9.9145 |  |  |  |
| Obese | G | 3.996528 | 8.355705 |  |  |  |
| Obese | G | 5.995139 | 6.035066 |  |  |  |
| Obese | G | 8.004861 | 4.448184 |  |  |  |
| Obese | G | 10.00069 | 3.177274 |  |  |  |
| Obese | G | 10.99375 | 3.043864 |  |  |  |
| Obese | G | 11.47292 | 2.871835 |  |  |  |
| Obese | G | 11.99583 | 2.741935 |  |  |  |
| Obese | G | 12.50139 | 2.554107 |  |  |  |
| Obese | G | 13.00139 | 2.401387 |  |  |  |
| Obese | G | 13.45 | 2.025732 |  |  |  |
| Obese | G | 13.99236 | 2.057329 |  |  |  |
| Obese | G | 14.47847 | 1.769443 |  |  |  |
| Obese | G | 14.99028 | 1.952005 |  |  |  |
| Obese | G | 18.99861 | 1.14101 |  |  |  |
| Obese | G | 21 | 0.775887 |  |  |  |
| Obese | G | 25.99861 | 0.39672 |  |  |  |
| Obese | G | 27 | 0.428318 |  |  |  |
| Obese | G | 27.99861 | 0.380922 |  |  |  |
| Obese | G | 29.00556 | 0.319483 |  |  |  |
| Obese | G | 29.99931 | 0.273842 |  |  |  |
| Obese | G | 31.02569 | 0.231713 |  |  |  |
| Lean | H | 0 |  | 655 | 9.96 | 28.47 |
| Lean | H | 0.004161 | 265.8239 |  |  |  |
| Lean | H | 0.011094 | 113.8298 |  |  |  |
| Lean | H | 0.0208 | 94.1478 |  |  |  |
| Lean | H | 0.0417 | 63.47726 |  |  |  |
| Lean | H | 0.08335 | 46.47662 |  |  |  |
| Lean | H | 0.1667 | 31.87535 |  |  |  |
| Lean | H | 0.3333 | 21.38239 |  |  |  |
| Lean | H | 0.497222 | 15.73944 |  |  |  |
| Lean | H | 0.995139 | 9.091618 |  |  |  |
| Lean | H | 2.018056 | 5.338762 |  |  |  |
| Lean | H | 3.002778 | 3.229917 |  |  |  |
| Lean | H | 3.9875 | 2.642038 |  |  |  |
| Lean | H | 5.990278 | 1.377414 |  |  |  |
| Lean | H | 8.004167 | 0.861311 |  |  |  |
| Lean | H | 10.11944 | 0.464835 |  |  |  |
| Lean | H | 10.97569 | 0.444327 |  |  |  |
| Lean | H | 11.48542 | 0.384514 |  |  |  |
| Lean | H | 11.97917 | 0.321283 |  |  |  |
| Lean | H | 12.45347 | 0.317865 |  |  |  |
| Lean | H | 12.99861 | 0.252925 |  |  |  |
| Lean | H | 13.44444 | 0.191402 |  |  |  |
| Lean | H | 13.98611 | 0.225581 |  |  |  |
| Lean | H | 14.47292 | 0.232417 |  |  |  |
| Lean | H | 14.98264 | 0.152097 |  |  |  |
| Lean | H | 17.99028 | 0.054686 |  |  |  |
| Obese | H | 0 |  | 1035 | 15.1 | 38.7 |
| Obese | H | 0.003467 | 335.9858 |  |  |  |
| Obese | H | 0.011789 | 174.2604 |  |  |  |
| Obese | H | 0.0208 | 144.0817 |  |  |  |
| Obese | H | 0.0417 | 101.4418 |  |  |  |
| Obese | H | 0.08335 | 71.48784 |  |  |  |
| Obese | H | 0.167394 | 51.10889 |  |  |  |
| Obese | H | 0.331911 | 31.01786 |  |  |  |
| Obese | H | 0.488194 | 23.88312 |  |  |  |
| Obese | H | 0.996528 | 12.86149 |  |  |  |
| Obese | H | 1.990278 | 10.53708 |  |  |  |
| Obese | H | 3.005556 | 8.123248 |  |  |  |
| Obese | H | 3.999306 | 4.717071 |  |  |  |
| Obese | H | 5.994444 | 4.717071 |  |  |  |
| Obese | H | 8.004861 | 2.954885 |  |  |  |
| Obese | H | 9.999306 | 2.353163 |  |  |  |
| Obese | H | 10.98889 | 1.94127 |  |  |  |
| Obese | H | 11.46806 | 1.883963 |  |  |  |
| Obese | H | 11.99514 | 1.905453 |  |  |  |
| Obese | H | 12.46319 | 1.676226 |  |  |  |
| Obese | H | 13.00069 | 1.461325 |  |  |  |
| Obese | H | 13.44931 | 1.278659 |  |  |  |
| Obese | H | 13.99236 | 1.174791 |  |  |  |
| Obese | H | 14.47778 | 1.124647 |  |  |  |
| Obese | H | 14.99028 | 0.967053 |  |  |  |
| Obese | H | 18.99792 | 0.472782 |  |  |  |
| Obese | H | 21 | 0.351005 |  |  |  |
| Obese | H | 25.99722 | 0.12894 |  |  |  |
| Obese | H | 27 | 0.179084 |  |  |  |
| Obese | H | 27.99792 | 0.123568 |  |  |  |
| Obese | H | 29.00486 | 0.136104 |  |  |  |

**Supplementary Table S2: Moxidectin raw data**

| Status | Dogs | Time | Cobs | dose_total | Body Weight | % Body Fat |
| --- | --- | --- | --- | --- | --- | --- |
|  | Units | day | ug/L | ug in toto | Kg |  |
| Lean | A | 0 |  | 694 | 10.3 | 20.58 |
| Lean | A | 0.003472 | 238.8711 |  |  |  |
| Lean | A | 0.011111 | 98.06936 |  |  |  |
| Lean | A | 0.020833 | 82.41617 |  |  |  |
| Lean | A | 0.041667 | 52.82628 |  |  |  |
| Lean | A | 0.084722 | 43.09108 |  |  |  |
| Lean | A | 0.170139 | 32.7154 |  |  |  |
| Lean | A | 0.334689 | 19.29506 |  |  |  |
| Lean | A | 0.501389 | 13.61391 |  |  |  |
| Lean | A | 1.001389 | 7.168183 |  |  |  |
| Lean | A | 2.00625 | 5.618237 |  |  |  |
| Lean | A | 3.006944 | 4.034985 |  |  |  |
| Lean | A | 4.004861 | 3.154974 |  |  |  |
| Lean | A | 6.002778 | 2.882132 |  |  |  |
| Lean | A | 8.013194 | 2.887815 |  |  |  |
| Lean | A | 10.14583 | 2.660969 |  |  |  |
| Lean | A | 11 | 2.486087 |  |  |  |
| Lean | A | 11.51111 | 2.385436 |  |  |  |
| Lean | A | 12.00208 | 2.367822 |  |  |  |
| Lean | A | 12.46458 | 2.430729 |  |  |  |
| Lean | A | 13.02083 | 2.606869 |  |  |  |
| Lean | A | 13.45625 | 2.569125 |  |  |  |
| Lean | A | 14.00139 | 2.767911 |  |  |  |
| Lean | A | 14.50278 | 2.482313 |  |  |  |
| Lean | A | 15.00347 | 2.239492 |  |  |  |
| Lean | A | 18.00972 | 1.864566 |  |  |  |
| Lean | A | 21.00625 | 1.477058 |  |  |  |
| Lean | A | 28.00625 | 0.944864 |  |  |  |
| Lean | A | 35.03194 | 0.669331 |  |  |  |
| Lean | A | 42.00278 | 0.629071 |  |  |  |
| Lean | A | 53.01389 | 0.28434 |  |  |  |
| Obese | A | 0 |  | 1136 | 16.5 | 34.53 |
| Obese | A | 0.003467 | 295.515 |  |  |  |
| Obese | A | 0.0104 | 169.9317 |  |  |  |
| Obese | A | 0.020106 | 115.8894 |  |  |  |
| Obese | A | 0.0417 | 96.19806 |  |  |  |
| Obese | A | 0.08335 | 62.86027 |  |  |  |
| Obese | A | 0.167394 | 45.38003 |  |  |  |
| Obese | A | 0.335383 | 29.92617 |  |  |  |
| Obese | A | 0.5 | 15.94726 |  |  |  |
| Obese | A | 1.004861 | 6.905025 |  |  |  |
| Obese | A | 2 | 3.740112 |  |  |  |
| Obese | A | 3.006944 | 3.622698 |  |  |  |
| Obese | A | 4.002083 | 2.907657 |  |  |  |
| Obese | A | 6.004861 | 2.97362 |  |  |  |
| Obese | A | 8.010417 | 2.52507 |  |  |  |
| Obese | A | 10.00556 | 2.74143 |  |  |  |
| Obese | A | 10.99653 | 2.277049 |  |  |  |
| Obese | A | 11.47778 | 2.435762 |  |  |  |
| Obese | A | 12.00694 | 2.376629 |  |  |  |
| Obese | A | 12.52778 | 1.673855 |  |  |  |
| Obese | A | 13.01042 | 1.854116 |  |  |  |
| Obese | A | 13.46181 | 2.026652 |  |  |  |
| Obese | A | 13.99792 | 2.625112 |  |  |  |
| Obese | A | 14.47292 | 1.828365 |  |  |  |
| Obese | A | 14.99792 | 1.820639 |  |  |  |
| Obese | A | 19.00486 | 1.792312 |  |  |  |
| Obese | A | 21.00903 | 1.614626 |  |  |  |
| Obese | A | 26.00417 | 1.745959 |  |  |  |
| Obese | A | 27.00625 | 1.488443 |  |  |  |
| Obese | A | 28.00625 | 1.49 |  |  |  |
| Obese | A | 29.00625 | 1.491018 |  |  |  |
| Obese | A | 30.00903 | 1.313332 |  |  |  |
| Obese | A | 31.025 | 1.388012 |  |  |  |
| Obese | A | 35.01319 | 1.305607 |  |  |  |
| Obese | A | 41.00417 | 1.094444 |  |  |  |
| Obese | A | 53.01042 | 0.893581 |  |  |  |
| Lean | B | 0 |  | 600 | 9.25 | 15.85 |
| Lean | B | 0.003467 | 169.7004 |  |  |  |
| Lean | B | 0.0104 | 102.641 |  |  |  |
| Lean | B | 0.0208 | 73.18884 |  |  |  |
| Lean | B | 0.0417 | 50.33184 |  |  |  |
| Lean | B | 0.1667 | 29.91791 |  |  |  |
| Lean | B | 0.3333 | 17.04929 |  |  |  |
| Lean | B | 0.499306 | 12.68605 |  |  |  |
| Lean | B | 1 | 7.17394 |  |  |  |
| Lean | B | 2.003472 | 4.918421 |  |  |  |
| Lean | B | 3.011111 | 4.93581 |  |  |  |
| Lean | B | 4.004167 | 4.478744 |  |  |  |
| Lean | B | 6.002083 | 3.805566 |  |  |  |
| Lean | B | 8.011111 | 3.577034 |  |  |  |
| Lean | B | 10.14236 | 3.338565 |  |  |  |
| Lean | B | 10.99653 | 3.368373 |  |  |  |
| Lean | B | 11.50694 | 3.164681 |  |  |  |
| Lean | B | 11.99861 | 3.159713 |  |  |  |
| Lean | B | 12.46458 | 3.09016 |  |  |  |
| Lean | B | 13.01736 | 2.967199 |  |  |  |
| Lean | B | 13.45347 | 2.687743 |  |  |  |
| Lean | B | 13.99931 | 2.650483 |  |  |  |
| Lean | B | 14.45625 | 2.394625 |  |  |  |
| Lean | B | 15.00069 | 2.506298 |  |  |  |
| Lean | B | 18.00694 | 1.889818 |  |  |  |
| Lean | B | 21.00278 | 1.389097 |  |  |  |
| Lean | B | 28.00347 | 0.681088 |  |  |  |
| Lean | B | 35.03056 | 0.489953 |  |  |  |
| Lean | B | 42.00069 | 0.253053 |  |  |  |
| Lean | B | 53.00903 | 0.126526 |  |  |  |
| Obese | B | 0 |  | 1277 | 18.8 | 40.96 |
| Obese | B | 0.003467 | 432.1793 |  |  |  |
| Obese | B | 0.009706 | 198.9227 |  |  |  |
| Obese | B | 0.0208 | 151.8314 |  |  |  |
| Obese | B | 0.0417 | 131.5215 |  |  |  |
| Obese | B | 0.08335 | 76.43248 |  |  |  |
| Obese | B | 0.1667 | 56.96263 |  |  |  |
| Obese | B | 0.332606 | 28.71584 |  |  |  |
| Obese | B | 0.498611 | 12.85535 |  |  |  |
| Obese | B | 1.002083 | 7.80172 |  |  |  |
| Obese | B | 1.998611 | 5.085428 |  |  |  |
| Obese | B | 3.009722 | 4.55277 |  |  |  |
| Obese | B | 3.999306 | 3.765708 |  |  |  |
| Obese | B | 6.002083 | 3.829309 |  |  |  |
| Obese | B | 8.009028 | 3.27015 |  |  |  |
| Obese | B | 10.00347 | 3.596105 |  |  |  |
| Obese | B | 10.99653 | 3.389402 |  |  |  |
| Obese | B | 11.47569 | 3.088453 |  |  |  |
| Obese | B | 12.00347 | 2.897283 |  |  |  |
| Obese | B | 12.51944 | 2.702151 |  |  |  |
| Obese | B | 13.00833 | 2.523864 |  |  |  |
| Obese | B | 13.46042 | 2.579579 |  |  |  |
| Obese | B | 13.99444 | 2.5949 |  |  |  |
| Obese | B | 14.47431 | 2.576793 |  |  |  |
| Obese | B | 14.99514 | 2.44865 |  |  |  |
| Obese | B | 19.00278 | 2.384578 |  |  |  |
| Obese | B | 21.00694 | 2.535007 |  |  |  |
| Obese | B | 26.00208 | 2.306578 |  |  |  |
| Obese | B | 27.00347 | 2.089292 |  |  |  |
| Obese | B | 28.00347 | 2.11297 |  |  |  |
| Obese | B | 29.00347 | 2.06422 |  |  |  |
| Obese | B | 30.00486 | 2.04472 |  |  |  |
| Obese | B | 31.02292 | 2.028006 |  |  |  |
| Obese | B | 35.01042 | 1.961148 |  |  |  |
| Obese | B | 41.00139 | 1.846934 |  |  |  |
| Obese | B | 53.00764 | 1.846934 |  |  |  |
| Lean | C | 0 |  | 803 | 11.6 | 28.405 |
| Lean | C | 0.002772 | 445.223 |  |  |  |
| Lean | C | 0.009706 | 186.1332 |  |  |  |
| Lean | C | 0.0208 | 129.5048 |  |  |  |
| Lean | C | 0.041006 | 84.2416 |  |  |  |
| Lean | C | 0.08335 | 82.72933 |  |  |  |
| Lean | C | 0.166006 | 53.91856 |  |  |  |
| Lean | C | 0.334689 | 31.2941 |  |  |  |
| Lean | C | 0.499306 | 20.4933 |  |  |  |
| Lean | C | 0.995833 | 10.61954 |  |  |  |
| Lean | C | 2.001389 | 5.426296 |  |  |  |
| Lean | C | 3.009028 | 4.08882 |  |  |  |
| Lean | C | 3.999306 | 3.555383 |  |  |  |
| Lean | C | 5.999306 | 3.244643 |  |  |  |
| Lean | C | 8.00625 | 2.946851 |  |  |  |
| Lean | C | 10.1375 | 2.954619 |  |  |  |
| Lean | C | 10.99167 | 2.726743 |  |  |  |
| Lean | C | 11.50278 | 2.740985 |  |  |  |
| Lean | C | 11.99375 | 2.884703 |  |  |  |
| Lean | C | 12.45972 | 2.633521 |  |  |  |
| Lean | C | 12.98681 | 2.819965 |  |  |  |
| Lean | C | 13.45 | 2.620574 |  |  |  |
| Lean | C | 13.99444 | 2.612805 |  |  |  |
| Lean | C | 14.45972 | 2.392698 |  |  |  |
| Lean | C | 14.99653 | 2.370687 |  |  |  |
| Lean | C | 18.00208 | 2.014631 |  |  |  |
| Lean | C | 20.99792 | 1.890335 |  |  |  |
| Lean | C | 27.99722 | 1.613258 |  |  |  |
| Lean | C | 35.02778 | 1.463067 |  |  |  |
| Lean | C | 41.99722 | 1.134201 |  |  |  |
| Lean | C | 53.00486 | 0.712112 |  |  |  |
| Obese | C | 0 |  | 1126 | 16.6 | 40.03 |
| Obese | C | 0.003467 | 643.5895 |  |  |  |
| Obese | C | 0.0104 | 271.7155 |  |  |  |
| Obese | C | 0.0208 | 196.8077 |  |  |  |
| Obese | C | 0.0417 | 142.6084 |  |  |  |
| Obese | C | 0.08335 | 99.81284 |  |  |  |
| Obese | C | 0.1667 | 68.45658 |  |  |  |
| Obese | C | 0.332606 | 38.06339 |  |  |  |
| Obese | C | 0.497222 | 21.51191 |  |  |  |
| Obese | C | 1.007639 | 9.767827 |  |  |  |
| Obese | C | 1.997917 | 5.890512 |  |  |  |
| Obese | C | 3.001389 | 5.385234 |  |  |  |
| Obese | C | 4.001389 | 4.073886 |  |  |  |
| Obese | C | 6.001389 | 3.395784 |  |  |  |
| Obese | C | 8.008333 | 3.527711 |  |  |  |
| Obese | C | 10.00278 | 3.073884 |  |  |  |
| Obese | C | 10.99583 | 3.088396 |  |  |  |
| Obese | C | 11.47639 | 2.63216 |  |  |  |
| Obese | C | 12.00278 | 2.929756 |  |  |  |
| Obese | C | 12.52292 | 2.716009 |  |  |  |
| Obese | C | 13.00764 | 2.830007 |  |  |  |
| Obese | C | 13.45903 | 2.767308 |  |  |  |
| Obese | C | 13.99722 | 2.852807 |  |  |  |
| Obese | C | 14.47708 | 2.63336 |  |  |  |
| Obese | C | 14.99514 | 2.790108 |  |  |  |
| Obese | C | 19.00278 | 2.724559 |  |  |  |
| Obese | C | 21.00625 | 2.752064 |  |  |  |
| Obese | C | 26.00278 | 2.681715 |  |  |  |
| Obese | C | 27.00278 | 2.588853 |  |  |  |
| Obese | C | 28.00208 | 2.577598 |  |  |  |
| Obese | C | 29.00278 | 2.608551 |  |  |  |
| Obese | C | 30.00556 | 2.535388 |  |  |  |
| Obese | C | 31.02361 | 2.495992 |  |  |  |
| Obese | C | 35.00972 | 2.349666 |  |  |  |
| Obese | C | 41.00139 | 2.130176 |  |  |  |
| Obese | C | 53.00833 | 1.603964 |  |  |  |
| Lean | E | 0 |  | 777 | 11.3 | 27.315 |
| Lean | E | 0.003467 | 373.177 |  |  |  |
| Lean | E | 0.0104 | 179.7023 |  |  |  |
| Lean | E | 0.0208 | 134.9624 |  |  |  |
| Lean | E | 0.042394 | 101.1828 |  |  |  |
| Lean | E | 0.084044 | 67.68891 |  |  |  |
| Lean | E | 0.167394 | 27.79431 |  |  |  |
| Lean | E | 0.333994 | 22.49719 |  |  |  |
| Lean | E | 0.502083 | 13.67904 |  |  |  |
| Lean | E | 1.002778 | 6.605822 |  |  |  |
| Lean | E | 2.000694 | 3.622297 |  |  |  |
| Lean | E | 3.005556 | 3.178273 |  |  |  |
| Lean | E | 3.995139 | 2.921207 |  |  |  |
| Lean | E | 5.994444 | 2.648561 |  |  |  |
| Lean | E | 8.003472 | 2.375915 |  |  |  |
| Lean | E | 10.13056 | 2.173378 |  |  |  |
| Lean | E | 10.98542 | 2.178571 |  |  |  |
| Lean | E | 11.49583 | 2.194151 |  |  |  |
| Lean | E | 11.9875 | 2.00979 |  |  |  |
| Lean | E | 12.45556 | 2.26426 |  |  |  |
| Lean | E | 13.00694 | 2.095479 |  |  |  |
| Lean | E | 13.44792 | 1.924102 |  |  |  |
| Lean | E | 13.99375 | 1.903329 |  |  |  |
| Lean | E | 14.45556 | 1.866976 |  |  |  |
| Lean | E | 14.99167 | 1.895539 |  |  |  |
| Lean | E | 17.99722 | 1.646262 |  |  |  |
| Lean | E | 20.99444 | 1.402179 |  |  |  |
| Lean | E | 27.99583 | 1.274945 |  |  |  |
| Lean | E | 35.02639 | 0.841308 |  |  |  |
| Lean | E | 41.99375 | 0.727056 |  |  |  |
| Lean | E | 52.99861 | 0.441427 |  |  |  |
| Obese | E | 0 |  | 1150 | 16.8 | 40.8 |
| Obese | E | 0.003467 | 348.9376 |  |  |  |
| Obese | E | 0.0104 | 277.4565 |  |  |  |
| Obese | E | 0.0208 | 185.6853 |  |  |  |
| Obese | E | 0.0417 | 132.9243 |  |  |  |
| Obese | E | 0.08335 | 98.55791 |  |  |  |
| Obese | E | 0.167394 | 62.98242 |  |  |  |
| Obese | E | 0.328439 | 28.53193 |  |  |  |
| Obese | E | 0.484028 | 16.52975 |  |  |  |
| Obese | E | 0.985417 | 7.569385 |  |  |  |
| Obese | E | 1.977083 | 4.909706 |  |  |  |
| Obese | E | 2.990278 | 4.065946 |  |  |  |
| Obese | E | 3.982639 | 3.74495 |  |  |  |
| Obese | E | 5.98125 | 3.191615 |  |  |  |
| Obese | E | 7.988194 | 3.411726 |  |  |  |
| Obese | E | 9.981944 | 2.626051 |  |  |  |
| Obese | E | 10.97778 | 2.525166 |  |  |  |
| Obese | E | 11.45556 | 2.272916 |  |  |  |
| Obese | E | 11.98194 | 2.34919 |  |  |  |
| Obese | E | 12.49097 | 2.339512 |  |  |  |
| Obese | E | 12.98681 | 2.205666 |  |  |  |
| Obese | E | 13.4375 | 2.127589 |  |  |  |
| Obese | E | 13.97569 | 2.057878 |  |  |  |
| Obese | E | 14.97639 | 1.968648 |  |  |  |
| Obese | E | 18.98403 | 1.848744 |  |  |  |
| Obese | E | 20.98542 | 1.83759 |  |  |  |
| Obese | E | 25.98333 | 1.56711 |  |  |  |
| Obese | E | 26.98333 | 1.647976 |  |  |  |
| Obese | E | 27.98125 | 1.466726 |  |  |  |
| Obese | E | 28.98194 | 1.33288 |  |  |  |
| Obese | E | 29.98472 | 1.313361 |  |  |  |
| Obese | E | 31.00694 | 1.424899 |  |  |  |
| Obese | E | 34.98889 | 1.187881 |  |  |  |
| Obese | E | 40.98125 | 1.04567 |  |  |  |
| Obese | E | 52.9875 | 0.77519 |  |  |  |
| Lean | F | 0 |  | 689 | 10.3 | 27.475 |
| Lean | F | 0.004161 | 262.8618 |  |  |  |
| Lean | F | 0.0104 | 119.4473 |  |  |  |
| Lean | F | 0.0208 | 93.10871 |  |  |  |
| Lean | F | 0.0417 | 59.96153 |  |  |  |
| Lean | F | 0.086822 | 43.86127 |  |  |  |
| Lean | F | 0.170172 | 28.93824 |  |  |  |
| Lean | F | 0.3333 | 16.02016 |  |  |  |
| Lean | F | 0.5 | 10.08785 |  |  |  |
| Lean | F | 1.000694 | 4.053587 |  |  |  |
| Lean | F | 2.017361 | 2.180927 |  |  |  |
| Lean | F | 3.004167 | 1.847173 |  |  |  |
| Lean | F | 3.993056 | 1.655417 |  |  |  |
| Lean | F | 5.990278 | 1.407833 |  |  |  |
| Lean | F | 8.006944 | 1.237922 |  |  |  |
| Lean | F | 10.12708 | 1.160248 |  |  |  |
| Lean | F | 10.98264 | 1.017038 |  |  |  |
| Lean | F | 11.49306 | 1.078934 |  |  |  |
| Lean | F | 11.98542 | 1.068011 |  |  |  |
| Lean | F | 12.45278 | 1.060729 |  |  |  |
| Lean | F | 13.00486 | 1.038883 |  |  |  |
| Lean | F | 13.44583 | 0.963637 |  |  |  |
| Lean | F | 13.99167 | 0.893246 |  |  |  |
| Lean | F | 14.45625 | 1.01461 |  |  |  |
| Lean | F | 14.98889 | 0.943005 |  |  |  |
| Lean | F | 17.99583 | 0.769453 |  |  |  |
| Lean | F | 20.99444 | 0.609252 |  |  |  |
| Lean | F | 27.99861 | 0.468468 |  |  |  |
| Lean | F | 35.025 | 0.354385 |  |  |  |
| Lean | F | 41.99236 | 0.288848 |  |  |  |
| Lean | F | 52.99653 | 0.138356 |  |  |  |
| Obese | F | 0 |  | 1001 | 15.1 | 35.38 |
| Obese | F | 0.003467 | 412.4318 |  |  |  |
| Obese | F | 0.0104 | 204.7698 |  |  |  |
| Obese | F | 0.0208 | 141.3215 |  |  |  |
| Obese | F | 0.0417 | 91.35942 |  |  |  |
| Obese | F | 0.08335 | 65.58944 |  |  |  |
| Obese | F | 0.162533 | 43.50208 |  |  |  |
| Obese | F | 0.329133 | 21.50752 |  |  |  |
| Obese | F | 0.490278 | 12.22063 |  |  |  |
| Obese | F | 1.000694 | 4.678739 |  |  |  |
| Obese | F | 1.993056 | 3.351637 |  |  |  |
| Obese | F | 3.00625 | 2.727617 |  |  |  |
| Obese | F | 3.997917 | 2.335134 |  |  |  |
| Obese | F | 5.996528 | 1.908767 |  |  |  |
| Obese | F | 8.005556 | 1.598169 |  |  |  |
| Obese | F | 9.997222 | 1.397692 |  |  |  |
| Obese | F | 10.99236 | 1.346867 |  |  |  |
| Obese | F | 11.46597 | 1.53605 |  |  |  |
| Obese | F | 11.99653 | 1.304513 |  |  |  |
| Obese | F | 12.50278 | 1.305924 |  |  |  |
| Obese | F | 13.00347 | 1.225451 |  |  |  |
| Obese | F | 13.45208 | 1.228275 |  |  |  |
| Obese | F | 13.99306 | 1.185921 |  |  |  |
| Obese | F | 14.47569 | 1.20851 |  |  |  |
| Obese | F | 14.99167 | 1.17745 |  |  |  |
| Obese | F | 19 | 1.101212 |  |  |  |
| Obese | F | 21.00139 | 1.092741 |  |  |  |
| Obese | F | 25.99931 | 0.861204 |  |  |  |
| Obese | F | 26.99931 | 0.835792 |  |  |  |
| Obese | F | 27.99861 | 0.769437 |  |  |  |
| Obese | F | 29.00347 | 0.753907 |  |  |  |
| Obese | F | 30 | 0.796261 |  |  |  |
| Obese | F | 31.02639 | 0.736965 |  |  |  |
| Obese | F | 35.00417 | 0.731318 |  |  |  |
| Obese | F | 40.99722 | 0.539312 |  |  |  |
| Obese | F | 53.00486 | 0.516723 |  |  |  |
| Lean | G | 0 |  | 705 | 10.6 | 24.365 |
| Lean | G | 0.003467 | 304.2191 |  |  |  |
| Lean | G | 0.011094 | 153.9567 |  |  |  |
| Lean | G | 0.0208 | 117.9771 |  |  |  |
| Lean | G | 0.042394 | 74.41857 |  |  |  |
| Lean | G | 0.084044 | 54.92315 |  |  |  |
| Lean | G | 0.167394 | 36.9353 |  |  |  |
| Lean | G | 0.333994 | 20.4936 |  |  |  |
| Lean | G | 0.499306 | 13.05611 |  |  |  |
| Lean | G | 0.997222 | 6.091996 |  |  |  |
| Lean | G | 2.020833 | 3.268531 |  |  |  |
| Lean | G | 3.002778 | 3.043425 |  |  |  |
| Lean | G | 3.990278 | 2.420848 |  |  |  |
| Lean | G | 5.990972 | 2.112132 |  |  |  |
| Lean | G | 8.00625 | 1.926902 |  |  |  |
| Lean | G | 9.876389 | 1.888313 |  |  |  |
| Lean | G | 10.97917 | 1.749391 |  |  |  |
| Lean | G | 11.49028 | 1.66578 |  |  |  |
| Lean | G | 11.98194 | 1.674784 |  |  |  |
| Lean | G | 12.45556 | 1.615614 |  |  |  |
| Lean | G | 13.00208 | 1.63105 |  |  |  |
| Lean | G | 13.44375 | 1.587315 |  |  |  |
| Lean | G | 13.98889 | 1.517854 |  |  |  |
| Lean | G | 14.45417 | 1.656776 |  |  |  |
| Lean | G | 14.98611 | 1.772544 |  |  |  |
| Lean | G | 17.99375 | 1.20142 |  |  |  |
| Lean | G | 20.99167 | 0.936439 |  |  |  |
| Lean | G | 27.99653 | 0.58656 |  |  |  |
| Lean | G | 35.02361 | 0.43992 |  |  |  |
| Lean | G | 41.99375 | 0.319007 |  |  |  |
| Lean | G | 52.99514 | 0.108051 |  |  |  |
| Obese | G | 0 |  | 1125 | 17 | 38.78 |
| Obese | G | 0.003467 | 502.8193 |  |  |  |
| Obese | G | 0.0104 | 220.9933 |  |  |  |
| Obese | G | 0.0208 | 161.8179 |  |  |  |
| Obese | G | 0.0417 | 111.0526 |  |  |  |
| Obese | G | 0.084044 | 83.74409 |  |  |  |
| Obese | G | 0.167394 | 51.623 |  |  |  |
| Obese | G | 0.331911 | 23.81948 |  |  |  |
| Obese | G | 0.488889 | 14.78989 |  |  |  |
| Obese | G | 1.001389 | 6.206325 |  |  |  |
| Obese | G | 1.990972 | 3.759525 |  |  |  |
| Obese | G | 3.008333 | 3.011854 |  |  |  |
| Obese | G | 3.996528 | 2.67838 |  |  |  |
| Obese | G | 5.995139 | 2.249627 |  |  |  |
| Obese | G | 8.004861 | 1.929386 |  |  |  |
| Obese | G | 10.00069 | 1.619732 |  |  |  |
| Obese | G | 10.99375 | 1.577386 |  |  |  |
| Obese | G | 11.47292 | 1.553566 |  |  |  |
| Obese | G | 11.99583 | 1.582679 |  |  |  |
| Obese | G | 12.50139 | 1.55092 |  |  |  |
| Obese | G | 13.00139 | 1.458288 |  |  |  |
| Obese | G | 13.45 | 1.349776 |  |  |  |
| Obese | G | 13.99236 | 1.421235 |  |  |  |
| Obese | G | 14.47847 | 1.365656 |  |  |  |
| Obese | G | 14.99028 | 1.413295 |  |  |  |
| Obese | G | 18.99861 | 1.273024 |  |  |  |
| Obese | G | 21 | 1.138047 |  |  |  |
| Obese | G | 25.99861 | 1.228032 |  |  |  |
| Obese | G | 27 | 1.053355 |  |  |  |
| Obese | G | 27.99861 | 1.094378 |  |  |  |
| Obese | G | 29.00556 | 1.056002 |  |  |  |
| Obese | G | 29.99931 | 0.992483 |  |  |  |
| Obese | G | 31.02569 | 0.96337 |  |  |  |
| Obese | G | 35.00278 | 0.910437 |  |  |  |
| Obese | G | 40.99514 | 0.93161 |  |  |  |
| Obese | G | 53.00625 | 0.656362 |  |  |  |
| Lean | H | 0 |  | 655 | 9.96 | 28.47 |
| Lean | H | 0.004161 | 349.42 |  |  |  |
| Lean | H | 0.011094 | 134 |  |  |  |
| Lean | H | 0.0208 | 105.5679 |  |  |  |
| Lean | H | 0.0417 | 69.95256 |  |  |  |
| Lean | H | 0.08335 | 47.72302 |  |  |  |
| Lean | H | 0.1667 | 32.09262 |  |  |  |
| Lean | H | 0.3333 | 20.65568 |  |  |  |
| Lean | H | 0.497222 | 13.67627 |  |  |  |
| Lean | H | 0.995139 | 5.820138 |  |  |  |
| Lean | H | 2.018056 | 2.684288 |  |  |  |
| Lean | H | 3.002778 | 1.91716 |  |  |  |
| Lean | H | 3.9875 | 1.724387 |  |  |  |
| Lean | H | 5.990278 | 1.594992 |  |  |  |
| Lean | H | 8.004167 | 1.357328 |  |  |  |
| Lean | H | 10.11944 | 1.172478 |  |  |  |
| Lean | H | 10.97569 | 1.198885 |  |  |  |
| Lean | H | 11.48542 | 1.223972 |  |  |  |
| Lean | H | 11.97917 | 1.322999 |  |  |  |
| Lean | H | 12.45347 | 1.140789 |  |  |  |
| Lean | H | 12.99861 | 1.204166 |  |  |  |
| Lean | H | 13.44444 | 1.235855 |  |  |  |
| Lean | H | 13.98611 | 1.122304 |  |  |  |
| Lean | H | 14.47292 | 1.085334 |  |  |  |
| Lean | H | 14.98264 | 1.117023 |  |  |  |
| Lean | H | 17.99028 | 0.937454 |  |  |  |
| Lean | H | 20.98958 | 0.77373 |  |  |  |
| Lean | H | 27.99514 | 0.467407 |  |  |  |
| Lean | H | 35.02083 | 0.33273 |  |  |  |
| Lean | H | 41.99236 | 0.285197 |  |  |  |
| Lean | H | 52.99167 | 0.150521 |  |  |  |
| Obese | H | 0 |  | 1035 | 15.1 | 38.7 |
| Obese | H | 0.003467 | 380.5075 |  |  |  |
| Obese | H | 0.011789 | 173.158 |  |  |  |
| Obese | H | 0.0208 | 140.0557 |  |  |  |
| Obese | H | 0.0417 | 104.2748 |  |  |  |
| Obese | H | 0.08335 | 66.84247 |  |  |  |
| Obese | H | 0.167394 | 43.81926 |  |  |  |
| Obese | H | 0.331911 | 23.11241 |  |  |  |
| Obese | H | 0.488194 | 15.0551 |  |  |  |
| Obese | H | 0.996528 | 6.035554 |  |  |  |
| Obese | H | 1.990278 | 3.565112 |  |  |  |
| Obese | H | 3.005556 | 2.705498 |  |  |  |
| Obese | H | 3.999306 | 2.521842 |  |  |  |
| Obese | H | 5.994444 | 1.998286 |  |  |  |
| Obese | H | 8.004861 | 1.776254 |  |  |  |
| Obese | H | 9.999306 | 1.696761 |  |  |  |
| Obese | H | 10.98889 | 1.447318 |  |  |  |
| Obese | H | 11.46806 | 1.400719 |  |  |  |
| Obese | H | 11.99514 | 1.526811 |  |  |  |
| Obese | H | 12.46319 | 1.535034 |  |  |  |
| Obese | H | 13.00069 | 1.411683 |  |  |  |
| Obese | H | 13.44931 | 1.356861 |  |  |  |
| Obese | H | 13.99236 | 1.296556 |  |  |  |
| Obese | H | 14.47778 | 1.35412 |  |  |  |
| Obese | H | 14.99028 | 1.299297 |  |  |  |
| Obese | H | 18.99792 | 1.263662 |  |  |  |
| Obese | H | 21 | 1.126606 |  |  |  |
| Obese | H | 25.99722 | 1.01696 |  |  |  |
| Obese | H | 27 | 0.923762 |  |  |  |
| Obese | H | 27.99792 | 0.912797 |  |  |  |
| Obese | H | 29.00486 | 0.89635 |  |  |  |
| Obese | H | 29.99931 | 0.794929 |  |  |  |
| Obese | H | 31.02639 | 0.997772 |  |  |  |
| Obese | H | 35.00208 | 0.707212 |  |  |  |
| Obese | H | 40.99653 | 0.561932 |  |  |  |
| Obese | H | 53.00625 | 0.419393 |  |  |  |

**Supplementary Table S3: Eprinomectin raw data**

| Status | Dogs | Time | Cobs | dose_tot | Body Weight | %Body Fat |
| --- | --- | --- | --- | --- | --- | --- |
|  | Units | day | ug/L | ug | Kg |  |
| Lean | A | 0 |  | 694 | 10.3 | 20.58 |
| Lean | A | 0.003472 | 98.41625 |  |  |  |
| Lean | A | 0.011111 | 71.4573 |  |  |  |
| Lean | A | 0.020833 | 59.85101 |  |  |  |
| Lean | A | 0.041667 | 51.05861 |  |  |  |
| Lean | A | 0.084722 | 37.81638 |  |  |  |
| Lean | A | 0.170139 | 30.12066 |  |  |  |
| Lean | A | 0.334689 | 26.05619 |  |  |  |
| Lean | A | 0.501389 | 22.22731 |  |  |  |
| Lean | A | 1.001389 | 9.158368 |  |  |  |
| Lean | A | 2.00625 | 2.985949 |  |  |  |
| Lean | A | 3.006944 | 1.334744 |  |  |  |
| Lean | A | 4.004861 | 0.766601 |  |  |  |
| Lean | A | 6.002778 | 0.184017 |  |  |  |
| Obese | A | 0 |  | 1136 | 16.5 | 34.53 |
| Obese | A | 0.003467 | 243.856 |  |  |  |
| Obese | A | 0.0104 | 180.2487 |  |  |  |
| Obese | A | 0.020106 | 147.3743 |  |  |  |
| Obese | A | 0.0417 | 99.98012 |  |  |  |
| Obese | A | 0.08335 | 77.6798 |  |  |  |
| Obese | A | 0.167394 | 57.48574 |  |  |  |
| Obese | A | 0.335383 | 48.31387 |  |  |  |
| Obese | A | 0.5 | 44.75169 |  |  |  |
| Obese | A | 1.004861 | 16.96671 |  |  |  |
| Obese | A | 2 | 6.753032 |  |  |  |
| Obese | A | 3.006944 | 5.035299 |  |  |  |
| Obese | A | 4.002083 | 2.2 |  |  |  |
| Obese | A | 6.004861 | 0.479153 |  |  |  |
| Obese | A | 8.010417 | 0.333561 |  |  |  |
| Obese | A | 10.00556 | 0.26517 |  |  |  |
| Obese | A | 10.99653 | 0.328106 |  |  |  |
| Obese | A | 11.47778 | 0.20685 |  |  |  |
| Obese | A | 12.00694 | 0.209787 |  |  |  |
| Obese | A | 12.52778 | 0.659569 |  |  |  |
| Obese | A | 13.01042 | 0.779987 |  |  |  |
| Obese | A | 13.46181 | 0.286149 |  |  |  |
| Obese | A | 13.99792 | 0.350763 |  |  |  |
| Obese | A | 14.47292 | 0.279855 |  |  |  |
| Obese | A | 14.99792 | 0.302093 |  |  |  |
| Obese | A | 19.00486 | 0.130855 |  |  |  |
| Obese | A | 21.00903 | 0.161513 |  |  |  |
| Obese | A | 26.00417 | 0.259841 |  |  |  |
| Obese | A | 27.00625 | 0.121508 |  |  |  |
| Obese | A | 28.00625 | 0.163008 |  |  |  |
| Obese | A | 29.00625 | 0.057688 |  |  |  |
| Lean | B | 0 |  | 600 | 9.25 | 15.85 |
| Lean | B | 0.003467 | 84.91634 |  |  |  |
| Lean | B | 0.0104 | 70.13697 |  |  |  |
| Lean | B | 0.0208 | 55.13811 |  |  |  |
| Lean | B | 0.0417 | 45.46811 |  |  |  |
| Lean | B | 0.1667 | 41.96024 |  |  |  |
| Lean | B | 0.3333 | 34.2844 |  |  |  |
| Lean | B | 0.499306 | 18.7311 |  |  |  |
| Lean | B | 1 | 14.03552 |  |  |  |
| Lean | B | 2.003472 | 7.703076 |  |  |  |
| Lean | B | 3.011111 | 2.19455 |  |  |  |
| Lean | B | 4.004167 | 2.237636 |  |  |  |
| Lean | B | 6.002083 | 1.725479 |  |  |  |
| Lean | B | 8.011111 | 0.341031 |  |  |  |
| Lean | B | 10.14236 | 0.110561 |  |  |  |
| Obese | B | 0 |  | 1277 | 18.8 | 40.96 |
| Obese | B | 0.003467 | 335.2483 |  |  |  |
| Obese | B | 0.009706 | 227.2176 |  |  |  |
| Obese | B | 0.0208 | 186.6163 |  |  |  |
| Obese | B | 0.0417 | 148.2148 |  |  |  |
| Obese | B | 0.08335 | 105.4511 |  |  |  |
| Obese | B | 0.1667 | 77.28223 |  |  |  |
| Obese | B | 0.332606 | 56.92253 |  |  |  |
| Obese | B | 0.498611 | 61.47765 |  |  |  |
| Obese | B | 1.002083 | 28.46912 |  |  |  |
| Obese | B | 1.998611 | 14.51 |  |  |  |
| Obese | B | 3.009722 | 10.44791 |  |  |  |
| Obese | B | 3.999306 | 8.028658 |  |  |  |
| Obese | B | 6.002083 | 2.556833 |  |  |  |
| Obese | B | 8.009028 | 0.706597 |  |  |  |
| Obese | B | 10.00347 | 0.237775 |  |  |  |
| Obese | B | 10.99653 | 0.171228 |  |  |  |
| Obese | B | 11.47569 | 0.170854 |  |  |  |
| Obese | B | 12.00347 | 0.262076 |  |  |  |
| Obese | B | 12.51944 | 0.357785 |  |  |  |
| Obese | B | 13.00833 | 0.466952 |  |  |  |
| Obese | B | 13.46042 | 0.521909 |  |  |  |
| Obese | B | 13.99444 | 0.437043 |  |  |  |
| Obese | B | 14.47431 | 0.329371 |  |  |  |
| Obese | B | 14.99514 | 0.364888 |  |  |  |
| Obese | B | 19.00278 | 0.177963 |  |  |  |
| Obese | B | 21.00694 | 0.257598 |  |  |  |
| Obese | B | 26.00208 | 0.11777 |  |  |  |
| Obese | B | 27.00347 | 0.105058 |  |  |  |
| Lean | C | 0 |  | 803 | 11.6 | 28.405 |
| Lean | C | 0.002772 | 148.1006 |  |  |  |
| Lean | C | 0.009706 | 69.62498 |  |  |  |
| Lean | C | 0.0208 | 74.15042 |  |  |  |
| Lean | C | 0.041006 | 68.08591 |  |  |  |
| Lean | C | 0.08335 | 46.46809 |  |  |  |
| Lean | C | 0.166006 | 39.66826 |  |  |  |
| Lean | C | 0.334689 | 30.04097 |  |  |  |
| Lean | C | 0.499306 | 25.53222 |  |  |  |
| Lean | C | 0.995833 | 26.21458 |  |  |  |
| Lean | C | 2.001389 | 12.59197 |  |  |  |
| Lean | C | 3.009028 | 6.887422 |  |  |  |
| Lean | C | 3.999306 | 3.114835 |  |  |  |
| Lean | C | 5.999306 | 1.836034 |  |  |  |
| Lean | C | 8.00625 | 0.305728 |  |  |  |
| Lean | C | 10.1375 | 0.071323 |  |  |  |
| Obese | C | 0 |  | 1126 | 16.6 | 40.03 |
| Obese | C | 0.003467 | 373.1708 |  |  |  |
| Obese | C | 0.0104 | 247.6326 |  |  |  |
| Obese | C | 0.0208 | 207.0053 |  |  |  |
| Obese | C | 0.0417 | 149.7447 |  |  |  |
| Obese | C | 0.08335 | 101.3984 |  |  |  |
| Obese | C | 0.1667 | 83.40438 |  |  |  |
| Obese | C | 0.332606 | 71.29203 |  |  |  |
| Obese | C | 0.497222 | 61.67831 |  |  |  |
| Obese | C | 1.007639 | 33 |  |  |  |
| Obese | C | 1.997917 | 28.21992 |  |  |  |
| Obese | C | 3.001389 | 19.6 |  |  |  |
| Obese | C | 4.001389 | 11.5021 |  |  |  |
| Obese | C | 6.001389 | 4.658187 |  |  |  |
| Obese | C | 8.008333 | 2.778149 |  |  |  |
| Obese | C | 10.00278 | 1.472703 |  |  |  |
| Obese | C | 10.99583 | 1.431363 |  |  |  |
| Obese | C | 11.47639 | 1.477254 |  |  |  |
| Obese | C | 12.00278 | 1.052473 |  |  |  |
| Obese | C | 12.52292 | 0.80481 |  |  |  |
| Obese | C | 13.00764 | 0.862838 |  |  |  |
| Obese | C | 13.45903 | 0.72175 |  |  |  |
| Obese | C | 13.99722 | 0.717578 |  |  |  |
| Obese | C | 14.47708 | 0.62807 |  |  |  |
| Obese | C | 14.99514 | 0.731231 |  |  |  |
| Obese | C | 19.00278 | 0.516217 |  |  |  |
| Obese | C | 21.00625 | 0.273066 |  |  |  |
| Obese | C | 26.00278 | 0.235865 |  |  |  |
| Obese | C | 27.00278 | 0.050241 |  |  |  |
| Lean | E | 0 |  | 777 | 11.3 | 27.315 |
| Lean | E | 0.003467 | 194.3787 |  |  |  |
| Lean | E | 0.0104 | 125.9894 |  |  |  |
| Lean | E | 0.0208 | 89.91409 |  |  |  |
| Lean | E | 0.042394 | 69.67032 |  |  |  |
| Lean | E | 0.084044 | 57.49378 |  |  |  |
| Lean | E | 0.167394 | 44.31036 |  |  |  |
| Lean | E | 0.333994 | 36.52105 |  |  |  |
| Lean | E | 0.502083 | 35.52646 |  |  |  |
| Lean | E | 1.002778 | 16.98987 |  |  |  |
| Lean | E | 2.000694 | 6.24176 |  |  |  |
| Lean | E | 3.005556 | 2.628907 |  |  |  |
| Lean | E | 3.995139 | 0.967984 |  |  |  |
| Lean | E | 5.994444 | 0.160035 |  |  |  |
| Obese | E | 0 |  | 1150 | 16.8 | 40.8 |
| Obese | E | 0.003467 | 375.7442 |  |  |  |
| Obese | E | 0.0104 | 274.0206 |  |  |  |
| Obese | E | 0.0208 | 208.6465 |  |  |  |
| Obese | E | 0.0417 | 131.5525 |  |  |  |
| Obese | E | 0.08335 | 107.4352 |  |  |  |
| Obese | E | 0.167394 | 78.9 |  |  |  |
| Obese | E | 0.328439 | 65.52394 |  |  |  |
| Obese | E | 0.484028 | 57.46954 |  |  |  |
| Obese | E | 0.985417 | 32.1 |  |  |  |
| Obese | E | 1.977083 | 14.0646 |  |  |  |
| Obese | E | 2.990278 | 8.635112 |  |  |  |
| Obese | E | 3.982639 | 4.96346 |  |  |  |
| Obese | E | 5.98125 | 1.336919 |  |  |  |
| Obese | E | 7.988194 | 0.433916 |  |  |  |
| Obese | E | 9.981944 | 0.125396 |  |  |  |
| Obese | E | 10.97778 | 0.172037 |  |  |  |
| Obese | E | 11.45556 | 0.177772 |  |  |  |
| Obese | E | 11.98194 | 0.128837 |  |  |  |
| Obese | E | 12.49097 | 0.056581 |  |  |  |
| Obese | E | 12.98681 | 0.093282 |  |  |  |
| Obese | E | 13.4375 | 0.113162 |  |  |  |
| Obese | E | 13.97569 | 0.022556 |  |  |  |
| Obese | E | 14.97639 | 0.0929 |  |  |  |
| Obese | E | 18.98403 | 0.074549 |  |  |  |
| Obese | E | 20.98542 | 0.107272 |  |  |  |
| Obese | E | 25.98333 | 0.054926 |  |  |  |
| Obese | E | 26.98333 | 0.038706 |  |  |  |
| Lean | F | 0 |  | 689 | 10.3 | 27.475 |
| Lean | F | 0.004161 | 110.8197 |  |  |  |
| Lean | F | 0.0104 | 79.89852 |  |  |  |
| Lean | F | 0.0208 | 64.04505 |  |  |  |
| Lean | F | 0.0417 | 48.63043 |  |  |  |
| Lean | F | 0.086822 | 38.81197 |  |  |  |
| Lean | F | 0.170172 | 32.30172 |  |  |  |
| Lean | F | 0.3333 | 23.43581 |  |  |  |
| Lean | F | 0.5 | 17.42336 |  |  |  |
| Lean | F | 1.000694 | 9 |  |  |  |
| Lean | F | 2.017361 | 3.676436 |  |  |  |
| Lean | F | 3.004167 | 2.81208 |  |  |  |
| Lean | F | 3.993056 | 1.895479 |  |  |  |
| Lean | F | 5.990278 | 0.342733 |  |  |  |
| Lean | F | 8.006944 | 0.065621 |  |  |  |
| Obese | F | 0 |  | 1001 | 15.1 | 35.38 |
| Obese | F | 0.003467 | 272.0811 |  |  |  |
| Obese | F | 0.0104 | 199.0292 |  |  |  |
| Obese | F | 0.0208 | 151.2408 |  |  |  |
| Obese | F | 0.0417 | 116.5282 |  |  |  |
| Obese | F | 0.08335 | 94.71956 |  |  |  |
| Obese | F | 0.162533 | 66.7 |  |  |  |
| Obese | F | 0.329133 | 55.59595 |  |  |  |
| Obese | F | 0.490278 | 59.5532 |  |  |  |
| Obese | F | 1.000694 | 31.43801 |  |  |  |
| Obese | F | 1.993056 | 24.87298 |  |  |  |
| Obese | F | 3.00625 | 20.14356 |  |  |  |
| Obese | F | 3.997917 | 9.522434 |  |  |  |
| Obese | F | 5.996528 | 4.267169 |  |  |  |
| Obese | F | 8.005556 | 1.209294 |  |  |  |
| Obese | F | 9.997222 | 0.389729 |  |  |  |
| Obese | F | 10.99236 | 0.320453 |  |  |  |
| Obese | F | 11.46597 | 0.733679 |  |  |  |
| Obese | F | 11.99653 | 0.772571 |  |  |  |
| Obese | F | 12.50278 | 0.500328 |  |  |  |
| Obese | F | 13.00347 | 0.464095 |  |  |  |
| Obese | F | 13.45208 | 0.575066 |  |  |  |
| Obese | F | 13.99306 | 0.317278 |  |  |  |
| Obese | F | 14.47569 | 0.315753 |  |  |  |
| Obese | F | 14.99167 | 0.555618 |  |  |  |
| Obese | F | 19 | 0.133852 |  |  |  |
| Obese | F | 21.00139 | 0.40575 |  |  |  |
| Obese | F | 25.99931 | 0.269991 |  |  |  |
| Obese | F | 26.99931 | 0.346641 |  |  |  |
| Obese | F | 27.99861 | 0.277237 |  |  |  |
| Lean | G | 0 |  | 705 | 10.6 | 24.365 |
| Lean | G | 0.003467 | 124.1683 |  |  |  |
| Lean | G | 0.011094 | 90.42267 |  |  |  |
| Lean | G | 0.0208 | 79.60853 |  |  |  |
| Lean | G | 0.042394 | 57.10765 |  |  |  |
| Lean | G | 0.084044 | 47.94345 |  |  |  |
| Lean | G | 0.167394 | 41.09221 |  |  |  |
| Lean | G | 0.333994 | 32.54416 |  |  |  |
| Lean | G | 0.499306 | 26.65316 |  |  |  |
| Lean | G | 0.997222 | 12 |  |  |  |
| Lean | G | 2.020833 | 4.99249 |  |  |  |
| Lean | G | 3.002778 | 2.232941 |  |  |  |
| Lean | G | 3.990278 | 1.291752 |  |  |  |
| Lean | G | 5.990972 | 0.315508 |  |  |  |
| Lean | G | 8.00625 | 0.070875 |  |  |  |
| Obese | G | 0 |  | 1125 | 17 | 38.78 |
| Obese | G | 0.003467 | 320.3882 |  |  |  |
| Obese | G | 0.0104 | 241.0698 |  |  |  |
| Obese | G | 0.0208 | 162.7 |  |  |  |
| Obese | G | 0.0417 | 115.12 |  |  |  |
| Obese | G | 0.084044 | 93.37818 |  |  |  |
| Obese | G | 0.167394 | 70.83955 |  |  |  |
| Obese | G | 0.331911 | 60.92356 |  |  |  |
| Obese | G | 0.488889 | 55.6 |  |  |  |
| Obese | G | 1.001389 | 34.18107 |  |  |  |
| Obese | G | 1.990972 | 19.15531 |  |  |  |
| Obese | G | 3.008333 | 16.47276 |  |  |  |
| Obese | G | 3.996528 | 7.457939 |  |  |  |
| Obese | G | 5.995139 | 2.4 |  |  |  |
| Obese | G | 8.004861 | 0.7486 |  |  |  |
| Obese | G | 10.00069 | 0.169166 |  |  |  |
| Obese | G | 10.99375 | 0.169957 |  |  |  |
| Obese | G | 11.47292 | 0.305132 |  |  |  |
| Obese | G | 11.99583 | 0.364814 |  |  |  |
| Obese | G | 12.50139 | 0.374695 |  |  |  |
| Obese | G | 13.00139 | 0.116203 |  |  |  |
| Obese | G | 13.45 | 0.088536 |  |  |  |
| Obese | G | 13.99236 | 0.084188 |  |  |  |
| Obese | G | 14.47847 | 0.424949 |  |  |  |
| Obese | G | 14.99028 | 0.131294 |  |  |  |
| Obese | G | 18.99861 | 0.130924 |  |  |  |
| Obese | G | 21 | 0.172716 |  |  |  |
| Obese | G | 25.99861 | 0.053627 |  |  |  |
| Lean | H | 0 |  | 655 | 9.96 | 28.47 |
| Lean | H | 0.004161 | 114.0026 |  |  |  |
| Lean | H | 0.011094 | 90.98841 |  |  |  |
| Lean | H | 0.0208 | 73.45487 |  |  |  |
| Lean | H | 0.0417 | 55.90979 |  |  |  |
| Lean | H | 0.08335 | 39.54848 |  |  |  |
| Lean | H | 0.1667 | 30.8924 |  |  |  |
| Lean | H | 0.3333 | 21 |  |  |  |
| Lean | H | 0.497222 | 21.74052 |  |  |  |
| Lean | H | 0.995139 | 10.17307 |  |  |  |
| Lean | H | 2.018056 | 4.848821 |  |  |  |
| Lean | H | 3.002778 | 2 |  |  |  |
| Lean | H | 3.9875 | 0.855154 |  |  |  |
| Lean | H | 5.990278 | 0.248283 |  |  |  |
| Lean | H | 8.004167 | 0.083402 |  |  |  |
| Obese | H | 0 |  | 1035 | 15.1 | 38.7 |
| Obese | H | 0.003467 | 299.183 |  |  |  |
| Obese | H | 0.011789 | 154.2095 |  |  |  |
| Obese | H | 0.0208 | 145.7709 |  |  |  |
| Obese | H | 0.0417 | 135.5443 |  |  |  |
| Obese | H | 0.08335 | 93.39186 |  |  |  |
| Obese | H | 0.167394 | 64.95064 |  |  |  |
| Obese | H | 0.331911 | 52 |  |  |  |
| Obese | H | 0.488194 | 50.21596 |  |  |  |
| Obese | H | 0.996528 | 28.5558 |  |  |  |
| Obese | H | 1.990278 | 18.13699 |  |  |  |
| Obese | H | 3.005556 | 10.22457 |  |  |  |
| Obese | H | 3.999306 | 4.95198 |  |  |  |
| Obese | H | 5.994444 | 2.344332 |  |  |  |
| Obese | H | 8.004861 | 0.736577 |  |  |  |
| Obese | H | 9.999306 | 0.411259 |  |  |  |
| Obese | H | 10.98889 | 0.527416 |  |  |  |
| Obese | H | 11.46806 | 0.313546 |  |  |  |
| Obese | H | 11.99514 | 0.357497 |  |  |  |
| Obese | H | 12.46319 | 0.368485 |  |  |  |
| Obese | H | 13.00069 | 0.375548 |  |  |  |
| Obese | H | 13.44931 | 0.428525 |  |  |  |
| Obese | H | 13.99236 | 0.341015 |  |  |  |
| Obese | H | 14.47778 | 0.183654 |  |  |  |
| Obese | H | 14.99028 | 0.264885 |  |  |  |
| Obese | H | 18.99792 | 0.368229 |  |  |  |
| Obese | H | 21 | 0.209668 |  |  |  |
| Obese | H | 25.99722 | 0.231508 |  |  |  |
| Obese | H | 27 | 0.225393 |  |  |  |
| Obese | H | 27.99792 | 0.186517 |  |  |  |
| Obese | H | 29.00486 | 0.116191 |  |  |  |
